# Supplementary material for: First-tier detection of intragenomic 16S rRNA gene variation in culturable endophytic bacteria from cacao seeds
Source: PeerJ. 2019 Nov 20;7:e7452. doi: 10.7717/peerj.7452 (PMC6874854; doi:10.7717/peerj.7452)
Supplement: Supplemental Information 1 — P –Positive control: DNA template from amplifiable E. coli available bacterial isolate. N1 –Negative control without DNA sample. N2 –Negative control with E. coli DNA template but without Reverse primer. Beyond the controls, ladder and the indicated isolates shown in Fig. 1 (11, 17, 31, 45, 62 and 68), the remaining lanes of the two gels (a and b) shown in this picture contain amplifications for various other cacao endophytic bacterial isolates under study. These amplifications were repeated at least once, with similar amplicon patterns. The gels (c to g) were representatives of the experiments where the amplified fragments by the 799F/U1492R primers for each of the isolates under study (lanes) were subjected to Alu I digestion and electrophoresis on special 5–11% high-resolution polyacrilamyde-gradient. [file peerj-07-7452-s001.pdf]

_da Silva et al, PEERJ _SUPPL Mat--16S rDNA AMPLIFICATION [da Silva CB, et al. First-tier detection of intragenomic 16S rRNA gene variation in culturable endophytic bacteria from cacao seeds]

PCR products, amplified by 799F and U1492R primers, used to compose Figure 1.

P N1 N2 11 17 1 Kb 31

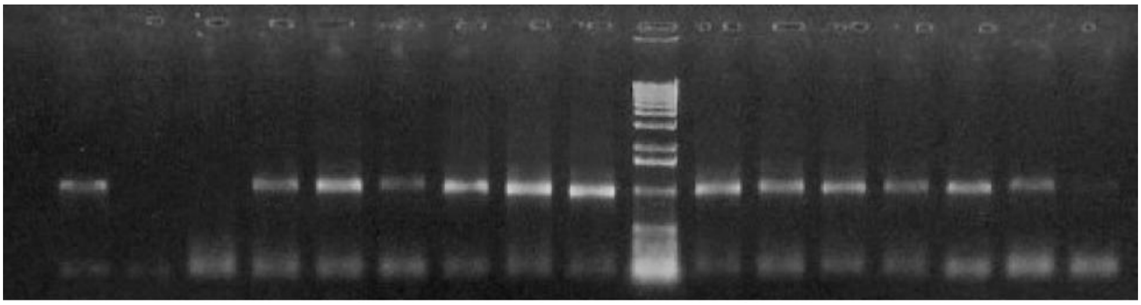

P N1 N2 45 62 1 Kb 68

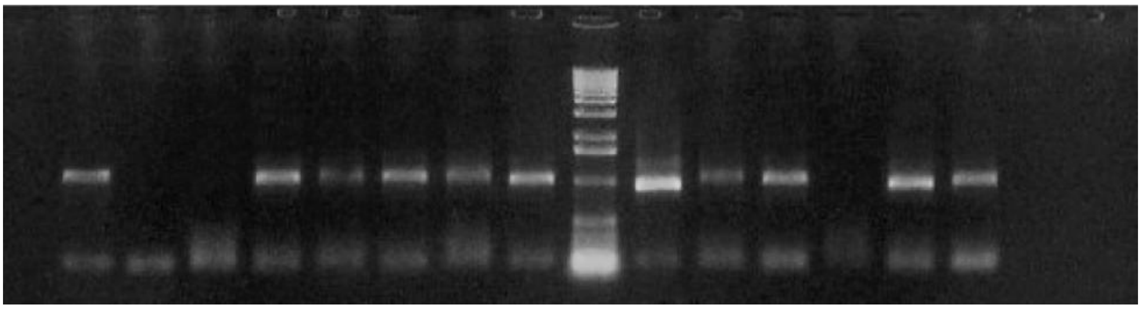

P – Positive control

N1 – Negative control without DNA sample

N2 – Negative control without Reverse primer

_da Silva et al, PEERJ _SUPPL Mat--RESTRICTION PROFILES OF BACTERIAL ENDOPHYTES [da Silva CB, et al. First-tier detection of intragenomic 16S rRNA gene variation in culturable endophytic bacteria from cacao seeds]

***Alu*I restriction profiles of bacterial endophytes from cacao, amplified by 799F and U1492R primers, used to compose Figure 1.**

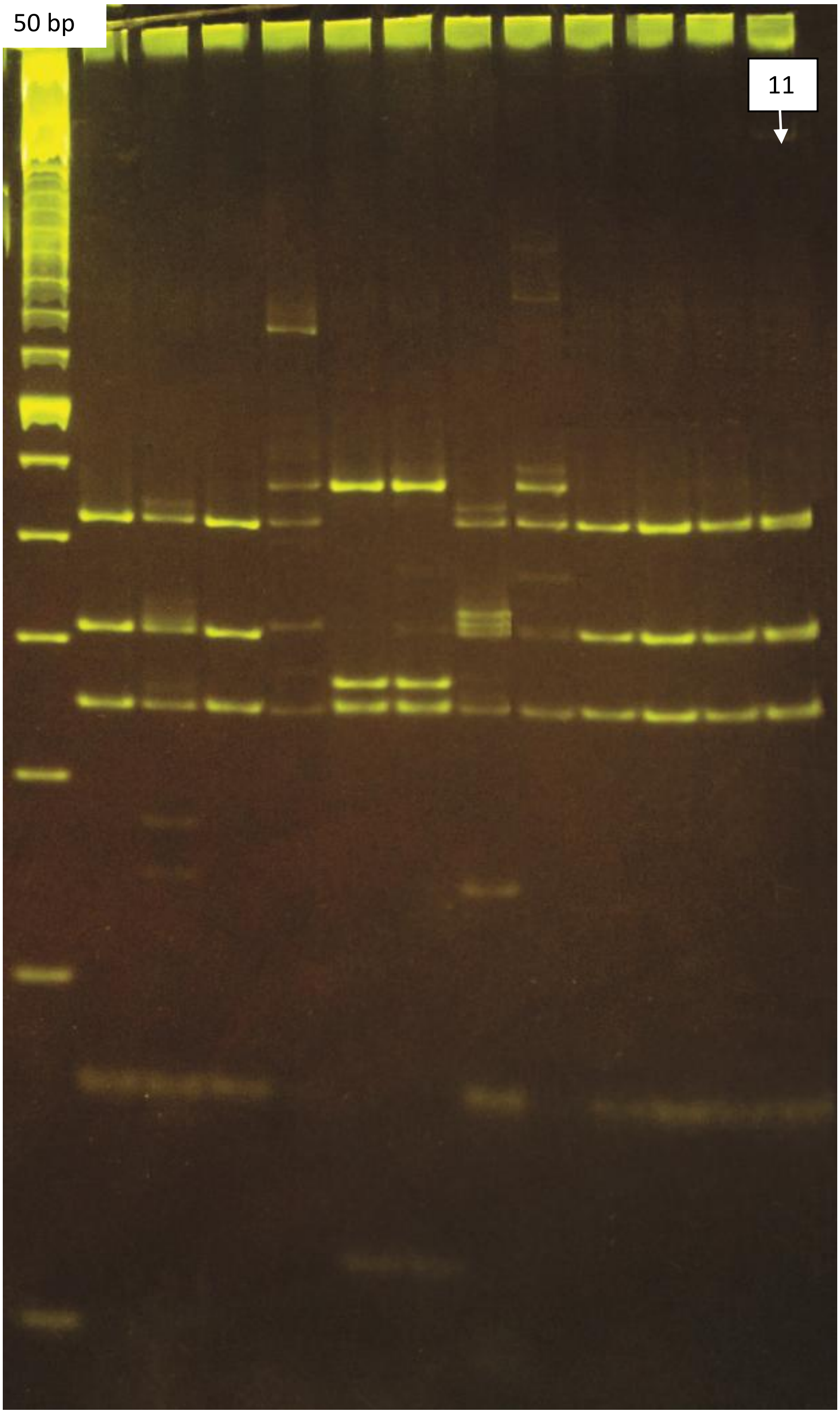

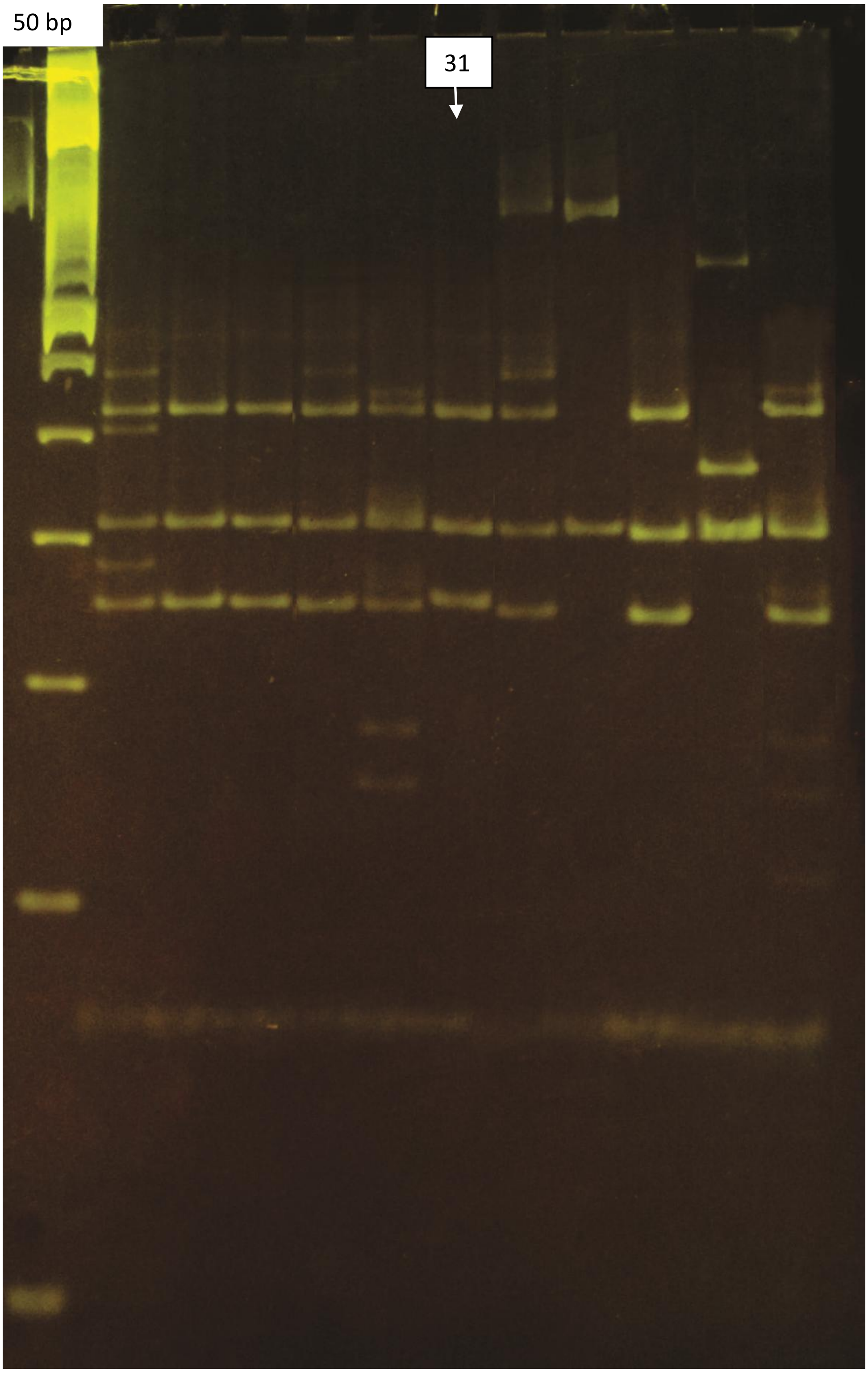
50 bp
31

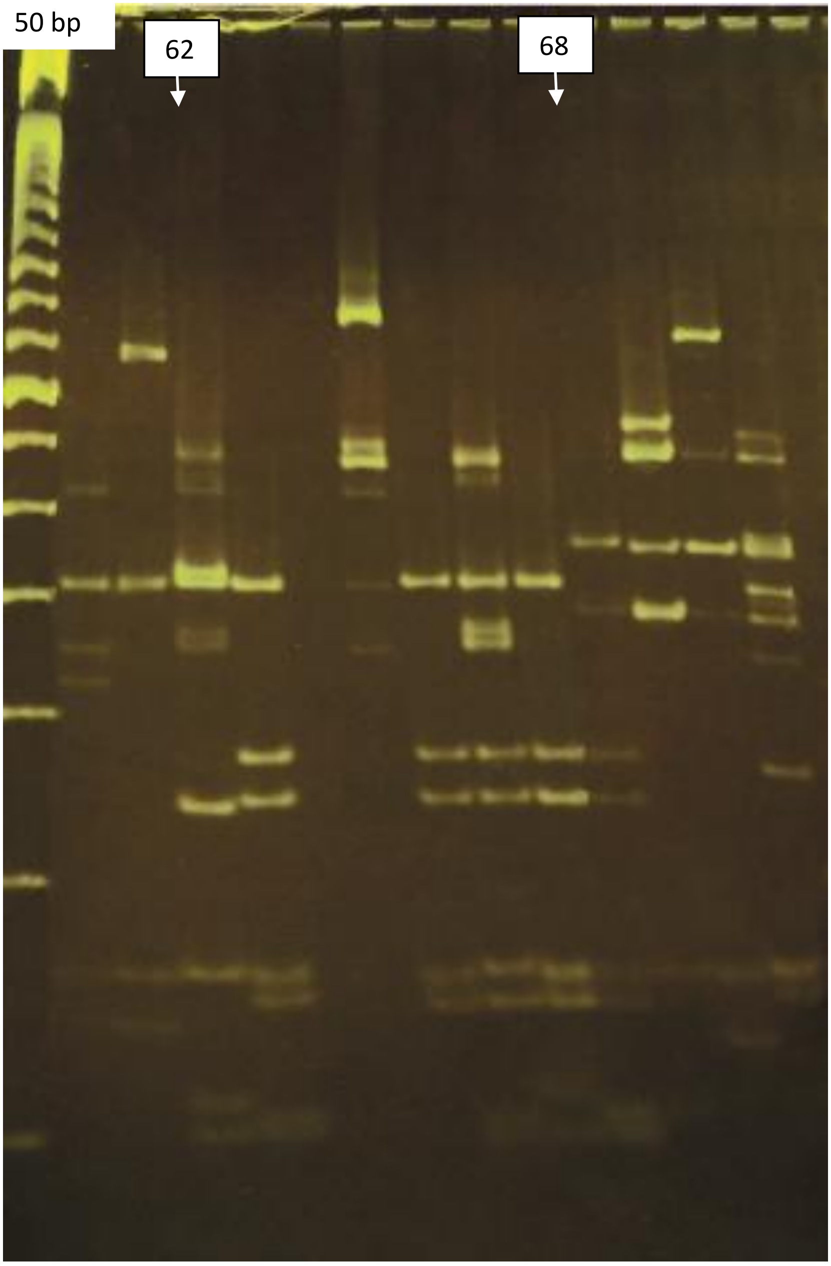
50 bp
62
68

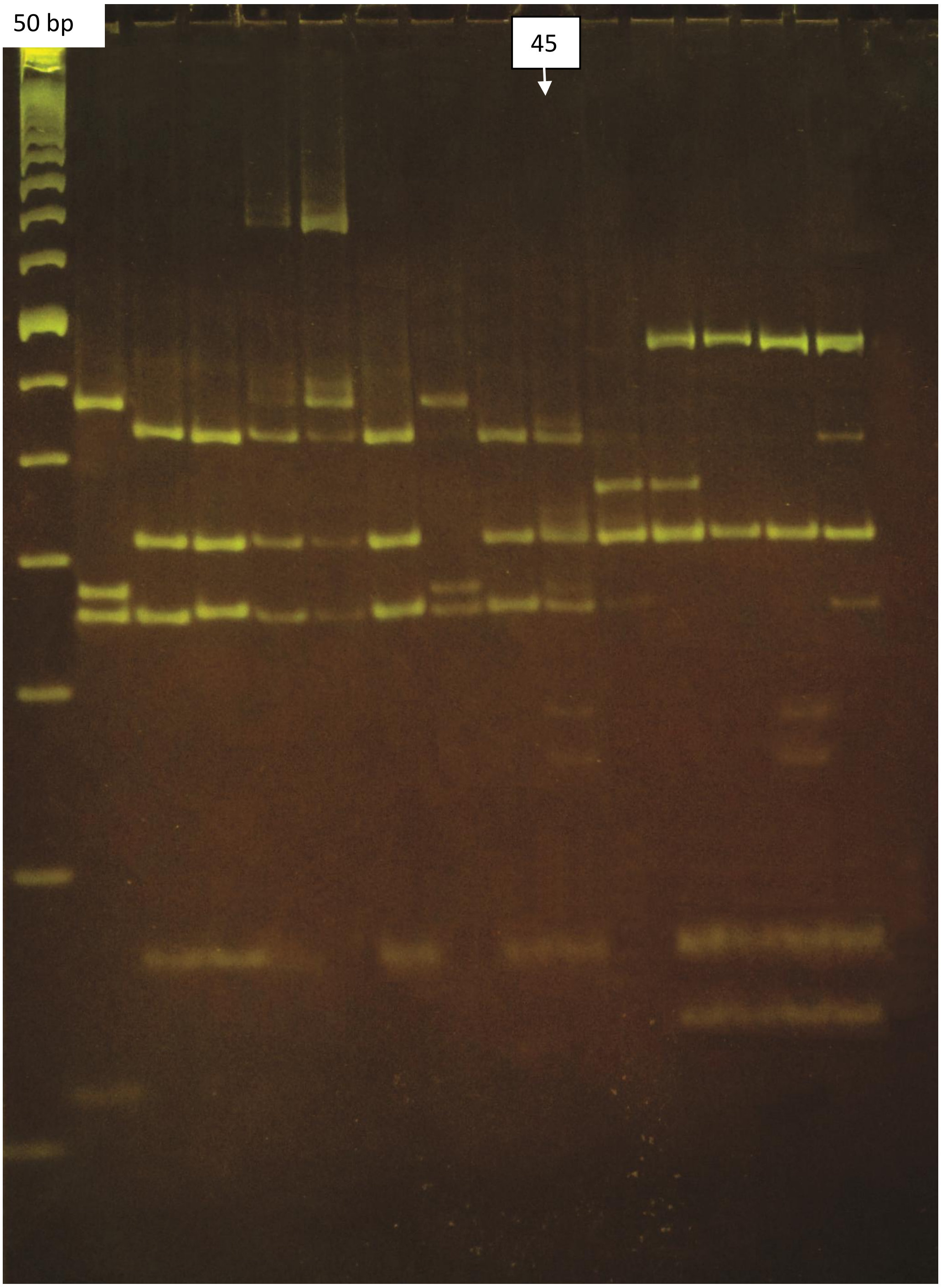
50 bp
45

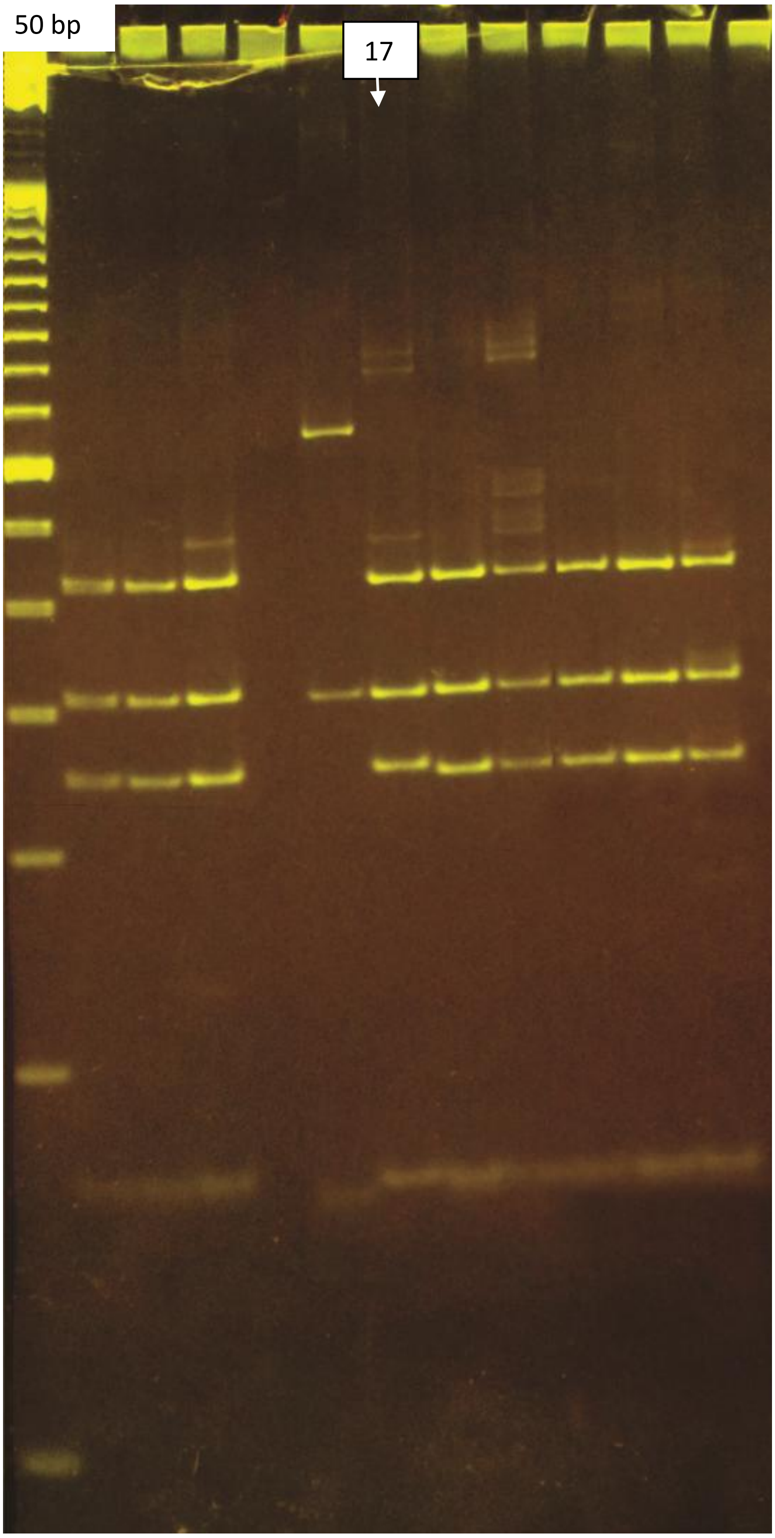
50 bp
17
