# Supplementary material for: First-tier detection of intragenomic 16S rRNA gene variation in culturable endophytic bacteria from cacao seeds
Source: PeerJ. 2019 Nov 20;7:e7452. doi: 10.7717/peerj.7452 (PMC6874854; doi:10.7717/peerj.7452)
Supplement: Supplemental Information 3 — Sequences are divided in the two groups of eletropherograms: clean and mixed. Order of isolates based on decreasing % sequence identity shown in Table 1. [file peerj-07-7452-s003.pdf]

_da Silva et al, PEERJ_SUPPL Mat--Multi-FASTA isol 16s rDNA SEQS

[da Silva, et al. First-tier detection of intragenomic 16S rRNA gene variation in culturable endophytic bacteria from cacao seeds]

Direct 16s rRNA gene amplicons Sanger sequencing of endophytic bacterial isolates from cacao seeds + adhered pulp.
Sequences are divided in the two groups of electropherograms: clean and mixed.
Order of isolates based on decreasing sequence identity shown in Table 1.

primer 799F
5'-AACMGGATTAGATACCCKG

primer U1492R
3'-AAGTCGTAACAAGGTAACC

_ _

"CLEAN ELECTROPHEROGRAMS"

100 % identity

>_isol_05
TGTTAGAGGGTTTCCGCCCTTTAGTGCTGAAGTTAACGCATTAAGCACTCCGCCTGGGGAGTACGGCCGCAAGGCT
GAAACTCAAAGGAATTGACGGGGGCCCGCACAAGCGGTGGAGCATGTGGTTTAATTCGAAGCAACGCGAAGAACCT
TACCAGGTCTTGACATCCTCTGACAACCCTAGAGATAGGGCTTCTCCTTCGGGAGCAGAGTGACAGGTGGTGCATG
GTTGTCGTCAGCTCGTGTCGTGAGATGTTGGGTTAAGTCCCGCAACGAGCGCAACCCTTGATCTTAGTTGCCATCA
TTAAGTTGGGCACTCTAAGGTGACTGCCGGTGACAAACCGGAGGAAGGTGGGGATGACGTCAAATCATCATGCCCC
TTATGACCTGGGCTACACACGTGCTACAATGGACGGTACAAAGAGCTGCAAGACCGCGAGGTGGAGCTAATCTCAT
AAAACCGTTCTCAGTTCGGATTGTAGGCTGCAACTCGCCTACATGAAGCTGGAATCGCTAGTAATCGCGGATCAGC
ATGCCGCGGTGAATACGTTCCCGGGCCTTGTACACACCGCCCGTCACACCACGAGAGTTTGTAACACCCGAAGTCG
GTGGGGTAACCTTTT

>_isol_06
TGTTAGAGGGTTTCCGCCCTTTAGTGCTGAAGTTAACGCATTAAGCACTCCGCCTGGGGAGTACGGCCGCAAGGCT
GAAACTCAAAGGAATTGACGGGGGCCCGCACAAGCGGTGGAGCATGTGGTTTAATTCGAAGCAACGCGAAGAACCT
TACCAGGTCTTGACATCCTCTGACAACCCTAGAGATAGGGCTTCTCCTTCGGGAGCAGAGTGACAGGTGGTGCATG
GTTGTCGTCAGCTCGTGTCGTGAGATGTTGGGTTAAGTCCCGCAACGAGCGCAACCCTTGATCTTAGTTGCCATCA
TTAAGTTGGGCACTCTAAGGTGACTGCCGGTGACAAACCGGAGGAAGGTGGGGATGACGTCAAATCATCATGCCCC
TTATGACCTGGGCTACACACGTGCTACAATGGACGGTACAAAGAGCTGCAAGACCGCGAGGTGGAGCTAATCTCAT
AAAACCGTTCTCAGTTCGGATTGTAGGCTGCAACTCGCCTACATGAAGCTGGAATCGCTAGTAATCGCGGATCAGC
ATGCCGCGGTGAATACGTTCCCGGGCCTTGTACACACCGCCCGTCACACCACGAGAGTTTGTAACACCCGAAGTCG
GTGGGGTAACCTTTT

>_isol_18
TGTTAGGGGGTTTCCGCCCCTTAGTGCTGCAGCTAACGCATTAAGCACTCCGCCTGGGGAGTACGGTCGCAAGACT
GAAACTCAAAGGAATTGACGGGGGCCCGCACAAGCGGTGGAGCATGTGGTTTAATTCGAAGCAACGCGAAGAACCT
TACCAGGTCTTGACATCCTCTGACAACCCTAGAGATAGGGCTTTCCCTTCGGGGACAGAGTGACAGGTGGTGCATG
GTTGTCGTCAGCTCGTGTCGTGAGATGTTGGGTTAAGTCCCGCAACGAGCGCAACCCTTGATCTTAGTTGCCAGCA
TTCAGTTGGGCACTCTAAGGTGACTGCCGGTGACAAACCGGAGGAAGGTGGGGATGACGTCAAATCATCATGCCCC
TTATGACCTGGGCTACACACGTGCTACAATGGACAGAACAAAGGGCTGCRAGACCGCAAGGTTTAGCCAATCCCAT
AAATCTGTTCTCAGTTCGGATCGCAGTCTGCAACTCGACTGCGTGAAGCTGGAATCGCTAGTAATCGCGGATCAGC
ATGCCGCGGTGAATACGTTCCCGGGCCTTGTACACACCGCCCGTCACACCACGAGAGTTTGCAACACCCGAAGTCG
GTGAGGTAACCTTTATGGAGCCAGCCGCCGAAGGTGGGGCAGATGATTGGGGTGAAGTCGTAACAAGGG

>_isol_31

TGTTAGGGGGTTTCCGCCCCTTAGTGCTGCAGCTAACGCATTAAGCACTCCGCCTGGGGAGTACGGTCGCAAGACT
GAAACTCAAAGGAATTGACGGGGGCCCGCACAAGCGGTGGAGCATGTGGTTTAATTCGAAGCAACGCGAAGAACCT
TACCAGGTCTTGACATCCTCTGACAACCCTAGAGATAGGGCTTTCCCTTCGGGGACAGAGTGACAGGTGGTGCATG
GTTGTCGTCAGCTCGTGTCGTGAGATGTTGGGTTAAGTCCCGCAACGAGCGCAACCCTTGATCTTAGTTGCCAGCA
TTCAGTTGGGCACTCTAAGGTGACTGCCGGTGACAAACCGGAGGAAGGTGGGGATGACGTCAAATCATCATGCCCC
TTATGACCTGGGCTACACACGTGCTACAATGGACAGAACAAAGGGCTGCRAGACCGCAAGGTTTAGCCAATCCCAT
AAATCTGTTCTCAGTTCGGATCGCAGTCTGCAACTCGACTGCGTGAAGCTGGAATCGCTAGTAATCGCGGATCAGC
ATGCCGCGGTGAATACGTTCCCGGGCCTTGTACACACCGCCCGTCACACCACGAGAGTTTGCAACACCCGAAGTCG
GTGAGGTAACCTTTATGGAGCCAGCCGCCGAAGGTGGGGCAGATGATTGGGGTGAAGTCGTAACAAGGTAACC

_ _

99 % identity (Bacillus spp)

>_isol_03
TGTTAGGGGGTTTCCGCCCCTTAGTGCTGCAGCTAACGCATTAAGCACTCCGCCTGGGGAGTACGGTCGCAAGACT
GAAACTCAAAGGAATTGACGGGGGCCCGCACAAGCGGTGGAGCATGTGGTTTAATTCGAAGCAACGCGAAGAACCT
TACCAGGTCTTGACATCCTCTGACAATCCTAGAGATAGGACGTCCCCTTCGGGGGCAGAGTGACAGGTGGTGCATG
GTTGTCGTCAGCTCGTGTCGTGAGATGTTGGGTTAAGTCCCGCAACGAGCGCAACCCTTGATCTTAGTTGCCAGCA
TTCAGTTGGGCACTCTAAGGTGACTGCCGGTGACAAACCGGAGGAAGGTGGGGATGACGTCAAATCATCATGCCCC
TTATGACCTGGGCTACACACGTGCTACAATGGACAGAACAAAGGGCAGCGAAACCGCGAGGTTAAGCCAATCCCAC
AAATCTGTTCTCAGTTCGGATCGCAGTCTGCAACTCGACTGCGTGAAGCTGGAATCGCTAGTAATCGCGGATCAGC
ATGCCGCGGTGAATACGTTCCCGGGCCTTGTACACACCGCCCGTCACACCACGAGAGTTTGTAACACCCGAAGTCG
GTGAGGTAACCTTTAWGGAGCCAGCCGCCGAAGGTGGGACAGATGATTGGGGTGAAGTCGTAACAAGGTAACC

>_isol_11
TGTTAGGGGGTTTCCGCCCCTTAGTGCTGCAGCTAACGCATTAAGCACTCCGCCTGGGGAGTACGGTCGCAAGACT
GAAACTCAAAGGAATTGACGGGGGCCCGCACAAGCGGTGGAGCATGTGGTTTAATTCGAAGCAACGCGAAGAACCT
TACCAGGTCTTGACATCCTCTGACAACCCTAGAGATAGGGCTTTCCCTTCGGGGACAGAGTGACAGGTGGTGCATG
GTTGTCGTCAGCTCGTGTCGTGAGATGTTGGGTTAAGTCCCGCAACGAGCGCAACCCTTGATCTTAGTTGCCAGCA
TTYAGTTGGGCACTCTAAGGTGACTGCCGGTGACAAACCGGAGGAAGGTGGGGATGACGTCAAATCATCATGCCCC
TTATGACCTGGGCTACACACGTGCTACAATGGACAGAACAAAGGGCTGCGAGACCGCAAGGTTTAGCCAATCCCAC
AAATCTGTTCTCAGTTCGGATCGCAGTCTGCAACTCGACTGCGTGAAGCTGGAATCGCTAGTAATCGCGGATCAGC
ATGCCGCGGTGAATACGTTCCCGGGCCTTGTACACACCGCCCGTCACACCACGAGAGTTTGCAACACCCGAAGTCG
GTGAGGTAACCTTTATGGAGCCAGCCGCCGAAGGTGGGGCAGATGATTGGGGTGAAGTCGTAACAAGGGTAGCC

>_isol_23
TGTTAGGGGGTTTCCGCCCCTTAGTGCTGCAGCTAACGCATTAAGCACTCCGCCTGGGGAGTACGGTCGCAAGACT
GAAACTCAAAGGAATTGACGGGGGCCCGCACAAGCGGTGGAGCATGTGGTTTAATTCGAAGCAACGCGAAGAACCT
TACCAGGTCTTGACATCCTCTGACAACCCTAGAGATAGGGCTTTCCCTTCGGGGACAGAGTGACAGGTGGTGCATG
GTTGTCGTCAGCTCGTGTCGTGAGATGTTGGGTTAAGTCCCGCAACGAGCGCAACCCTTGATCTTAGTTGCCAGCA
TTCAGTTGGGCACTCTAAGGTGACTGCCGGTGACAAACCGGAGGAAGGTGGGGATGACGTCAAATCATCATGCCCC
TTATGACCTGGGCTACACACGTGCTACAATGGACAGAACAAAGGGCTGCAAGACCGCAAGGTTTAGCCAATCCCAT
AAATCTGTTCTCAGTTCGGATCGCAGTCTGCAACTCGACTGCGTGAAGCTGGAATCGCTAGTAATCGCGGATCAGC
ATGCCGCGGTGAATACGTTCCCGGGCCTTGTACACACCGCCCGTCACACCACGAGAGTTTGCAACACCCGAAGTCG
GTGAGGTAACCTTTATGGAGCCAGCCGCCGAAGGTGGGGCAGATGATTGGGTGAAGTCGTACAAGGTAAC

>_isol_34
GTGTTAGGGGGTTTCCGCCCCTTAGTGCTGCAGCTAACGCATTAAGCACTCCGCCTGGGGAGTACGGTCGCAAGAC
TGAAACTCAAAGGAATTGACGGGGGCCCGCACAAGCGGTGGAGCATGTGGTTTAATTCGAAGCAACGCGAAGAACC
TTACCAGGTCTTGACATCCTCTGACAACCCTAGAGATAGGGCTTTCCCTTCGGGGACAGAGTGACAGGTGGTGCAT
GGTTGTCGTCAGCTCGTGTCGTGAGATGTTGGGTTAAGTCCCGCAACGAGCGCAACCCTTGATCTTAGTTGCCAGC
ATTCAGTTGGGCACTCTAAGGTGACTGCCGGTGACAAACCGGAGGAAGGTGGGGATGACGTCAAATCATCATGCCC
CTTATGACCTGGGCTACACACGTGCTACAATGGACAGAACAAAGGGCTGCRAGACCGCAAGGTTTAGCCAATCCCA
TAAATCTGTTCTCAGTTCGGATCGCAGTCTGCAACTCGACTGCGTGAAGCTGGAATCGCTAGTAATCGCGGATCAG
CATGCCGCGGTGAATACGTTCCCGGGCCTTGTACACACCGCCCGTCACACCACGAGAGTTTGCAACACCCGAAGTC

GGTGAGGTAACCTTTATGGAGCCAGCCGCCGAAGGTGGGGCAGATGATTGGGGTGAAGTCGTAACAAAGGTAACCC
AG

>_isol_37
TGTTAGAGGGTTTCCGCCCTTTAGTGCTGAAGTTAACGCATTAAGCACTCCGCCTGGGGAGTACGGCCGCAAGGCT
GAAACTCAAAGGAATTGACGGGGGCCCGCACAAGCGGTGGAGCATGTGGTTTAATTCGAAGCAACGCGAAGAACCT
TACCAGGTCTTGACATCCTCTGAAAACCCTAGAGATAGGGCTTCTCCTTCGGGAGCAGAGTGACAGGTGGTGCATG
GTTGTCGTCAGCTCGTGTCGTGAGATGTTGGGTTAAGTCCCGCAACGAGCGCAACCCTTGATCTTAGTTGCCATCA
TTAAGTTGGGCACTCTAAGGTGACTGCCGGTGACAAACCGGAGGAAGGTGGGGATGACGTCAAATCATCATGCCCC
TTATGACCTGGGCTACACACGTGCTACAATGGACGGTACAAAGAGCTGCAAGACCGCGAGGTGGAGCTAATCTCAT
AAAACCGTTCTCAGTTCGGATTGTAGGCTGCAACTCGCCTACATGAAGCTGGAATCGCTAGTAATCGCGGATCAGC
ATGCCGCGGTGAATACGTTCCCGGGCCTTGTACACACCGCCCGTCACACCACGAGAGTTTGTAACACCCGAAGTCG
GTGGGGTAACCTTTTTGGAGCCAGCCGCCTAAGGTGGGACAGATGATTGGGGTGAAGTCGTAACAAGGGTAG

>_isol_38
TGTTAGGGGGTTTCCGCCCCTTAGTGCTGCAGCTAACGCATTAAGCACTCCGCCTGGGGAGTACGGTCGCAAGACT
GAAACTCAAAGGAATTGACGGGGGCCCGCACAAGCGGTGGAGCATGTGGTTTAATTCGAAGCAACGCGAAGAACCT
TACCAGGTCTTGACATCCTCTGACAACCCTAGAGATAGGGCTTTCCCTTCGGGGACAGAGTGACAGGTGGTGCATG
GTTGTCGTCAGCTCGTGTCGTGAGATGTTGGGTTAAGTCCCGCAACGAGCGCAACCCTTGATCTTAGTTGCCAGCA
TTYAGTTGGGCACTCTAAGGTGACTGCCGGTGACAAACCGGAGGAAGGTGGGGATGACGTCAAATCATCATGCCCC
TTATGACCTGGGCTACACACGTGCTACAATGGACAGAACAAAGGGCTGCGAGACCGCAAGGTTTAGCCAATCCCAC
AAATCTGTTCTCAGTTCGGATCGCAGTCTGCAACTCGACTGCGTGAAGCTGGAATCGCTAGTAATCGCGGATCAGC
ATGCCGCGGTGAATACGTTCCCGGGCCTTGTACACACCGCCCGTCACACCACGAGAGTTTGCAACACCCGAAGTCG
GTGAGGTAACCTTTATGGAGCCAGCCGCCGAAGGTGGGGCAGATGATTGGGGTGAAGTCGTAACAAGG

>_isol_39
TGTTAGGGGGTTTCCGCCCCTTAgTGCTGCAGCTAACGCATTAAGCACTCCGCCTGGGgAGaACGGKCGCAAGACT
GAAACTCAAAGGAATTGACGGGGGCCCGcAcAagcGGTGGAGCATGTGGTTTAATTCGAAGCAACGCGAAGAACCT
TACCAGGTCTTGACATCCTCTGACAACCCTAgAGATAGGGCTTTCCCTTCGGGGACAGAGTGACAGGTGGTGCATG
GTTGTCGTCAGCTCGTGTCGTGAGATGTTGGGTTAAGTCCCGCAACGAGCGCAACCCTTGATCTTAGTTGCCAGCA
TTcAGTTGGGCACTCTAAGGTGACTGCCGGTGACAAACCGGAGGAAGGTGGGGATGACGTCAAATCATCATGCCCC
TTATGACCTGGGCTACACACGTGCTACAATGGACAGAACAAAGGGCTGCGAGACCGCAAGGTTTAGCCAATCCCAC
AAATCTGTTCTCAGTTCGGATCGCAGTCTGCAACTCGACTGCGTGAAGCTGGAATCGCTAGTAATCGCGGATCAGC
ATGCCGCGGTGAATACGTTCCCGGGCCTTGTACACACCGCCCGTCACACCACGAGAGTTTGCAACACCCGAAGTCG
GTGAGGTAACCTTTATGGAGCCAGCCGCCGAAGGTGGGGGCAGATGATTGGGGTGAAGTCGTAAC

_ _

99 % identity (other spp)

>_isol_53
TGTTGGGTACTTAGTTACTCAGTGTCGAAGCTAACGCGCTAAGCACACCGCCTGGGGAGTACGGCCGCAAGGTTGA
AACTCAAAGGAATTGACGGGGGCCCGCACAAGCGGTGGAGCATGTGGTTTAATTCGAAGCAACGCGCAGAACCTTA
CCAGGGCTTGCATGGGGAGGACGTACTCAGAGATGGGTATTTCTTCGGACCTCCCGCACAGGTGCTGCATGGCTGT
CGTCAGCTCGTGTCGTGAGATGTTGGGTTAAGTCCCGCAACGAGCGCAACCCTTGTCTTTAGTTGCCAGCACTTTC
AGGTGGGCACTCTAGAGAGACTGCCGGTGACAAGCCGGAGGAAGGTGGGGATGACGTCAAGTCCTCATGGCCCTTA
TGTCCTGGGCTACACACGTGCTACAATGGCGGTGACAGTGGGAAGCTATGTGGTGACACAGTGCTGATCTCTAAAA
GCCGTCTCAGTTCGGATTGTACTCTGCAACTCGAGTACATGAAGGTGGAATCGCTAGTAATCGCGGATCAGCATGC
CGCGGTGAATACGTTCCCGGGCCTTGTACACACCGCCCGTCACACCATGGGAGTTGGTTCGACCTTAAGCCGGTGA
GCGAACCGCAAGGACGCAGCCGACCACGGACGGGTCAGCGACTGGGGTGAAG

>_isol_57
TGTTAGGGGGTTTCCGCCCCTTAGTGCTGCAGCTAACGCATTAAGCACTCCGCCTGGGGAGTACGGTCGCAAGACT
GAAACTCAAAGGAATTGACGGGGGCCCGCACAAGCGGGGGAGCATGTGGTTTAATTCGAAGCAACGCGAAgAACCT
TACCAGGTCTTGACATCCCGTTGACCACTGTAgAgATATAGTTTCCCCTTCGGGGGCAACGGTGACAGGTGGTGCA
TGGTTGTCGTCAGCTCGTGTCGTGAGATGTTGGGTTAAGTCCCGCAACGAGCGCAACCCTTGATCTTAGTTGCCAT
CATTTAGTTGGGCACTCTAAGGTGACTGCCGGTGACAAACCGGAGGAAGGtGGGGATGACGTCAAATCATCATGCC

CCTTATGACCTGGGCTACACACGTGCTACAATGGACGATACAAACGGTTGCCAACTCGCGAGAGGGAGCTAATCCG
ATAAAGTCGTTCTCAGTTCGGATTGTAGGCTGCAACTCGCCTACATGAAGCCGGAATCGCTAGTAATCGCGGATCA
GCATGCCGCGGTGAATACGTTCCCGGGCCTTGTACACACCGCCCGTCACACCACGAGAGTTTGTAACACCCGAAGT
CGGTGAGGTAACCTTTTGGAGCCAGCCGCcGAAGGTggGATAGATGAtTGGGGTGAAGTCgTAACAAAGG

>_isol_62
TCGACTTGGAGGTTGTTCCCTTGAGGAGTGGCTTCCGGAGCTAACGCGTTAAGTCGACCGCCTGGGGAGTACGGCC
GCAAGGTTAAAACTCAAATGAATTGACGGGGGCCCGCACAAGCGGTGGAGCATGTGGTTTAATTCGATGCAACGCG
AAGAACCTTACCTACTCTTGACATCCAGAGAACTTWSCAGAGATGSWTTGGTGCCTTCGGGAACTCTGAGACAGGT
GCTGCATGGCTGTCGTCAGCTCGTGTTGTGAAATGTTGGGTTAAGTCCCGCAACGAGCGCAACCCTTATCCTTTGT
TGCCAGCGGTYCGGCCGGGAACTCAAAGGAGACTGCCAGTGATAAACTGGAGGAAGGTGGGGATGACGTCAAGTCA
TCATGGCCCTTACGAGTAGGGCTACACACGTGCTACAATGGCATATACAAAGAGAAGCGACCTCGCGAGAGCAAGC
GGACCTCATAAAGTATGTCGTAGTCCGGATTGGAGTCTGCAACTCGACTCCATGAAGTCGGAATCGCTAGTAATCG
TAGATCAGAATGCTACGGTGAATACGTTCCCGGGCCTTGTACACACCGCCCGTCACACCATGGGAGTGGGTTGCAA
AAGAAGTAGGTAGCTTAACCTTCGGGAGGGCGCTTACCACTTTGTGATTCATGACTGGGGTGAAGTCGTAACAAGG
TAACC

>_isol_67
TGTTAGGGGGTTTCCGCCCCTTAGTGCTGCAGCTAACGCATTAAGCACTCCGCCTGGGGAGTACGACCGCAAGGTT
GAAACTCAAAGGAATTGACGGGGACCCGCACAAGCGGTGGAGCATGTGGTTTAATTCGAAGCAACGCGAAGAACCT
TACCAAATCTTGACATCCTTTGACCGCTCTAGAGATAGAGTCTTCCCCTTCGGGGGACAAAGTGACAGGTGGTGCA
TGGTTGTCGTCAGCTCGTGTCGTGAGATGTTGGGTTAAGTCCCGCAACGAGCGCAACCCTTAAGCTTAGTTGCCAT
CATTAAGTTGGGCACTCTAAGTTGACTGCCGGTGACAAACCGGAGGAAGGTGGGGATGACGTCAAATCATCATGCC
CCTTATGATTTGGGCTACACACGTGCTACAATGGACAATACAAAGGGCAGCTAAACCGCGAGGTCAAGCAAATCCC
ATAAAGTTGTTCTCAGTTCGGATTGTAGTCTGCAACTCGACTACATGAAGCTGGAATCGCTAGTAATCGTAGATCA
GCATGCTACGGTGAATACGTTCCCGGGTCTTGTACACACCGCCCGTCACACCACGAGAGTTTGTAACACCCGAAGC
CGGTGGAGTAACYATTTATGGAGCTAGCCGTCGAAGGTGGGACAAATGATTGGGGTGAAGTCGTAACAAGGTAAMC

_ _

"MIXED ELECTROPHEROGRAMS"

99 % identity

>_isol_40
TGTTAGGGGGTTTCCGCCCCTTAGTGCTGCAGCTAACGCATTAAGCACTCCGCCTGGGGAGTACGGTCGCAAGACT
GAAACTCAAAGGAATTGACGGGGGCCCGCACAAGCGGTGGAGCATGTGGTTTAATTCGAAGCAACGCGAAGAACCT
TACCAGGTCTTGACATCCTCTGACAACCCTARARATAGGGCTTTCCCTTCGGGGACAGAGTGACAGGTGGTGCATG
GTTGTCGTCAGCTCGTGTCGTGAGATGTTGGGTTAAGTCCCGCAACGAGCGCAACCCTTGATCTTAGTTGCCAGCA
TTYAGTTGGGCACTCTAAGGTGACTGSCGGTGACAAACCGGARGAAGGKGGGGATGACGTCAAATCATCATGCCCC
TTATGACCTGGGCTACaCACGTGCTAcAATGGACAGAACAAAGGGCTGCGAgACCGCAAGGTTTARCCAATCCCAC
AAATCTGTTCTCAGTTCGGATCGCAgTCTGCAACTCGACTGCGTGAAGCTGGAATCGCTAGTAATCGCGGATCARC
ATGSCGCGGTGAATACGTTCCCGGGCCTTGTACACACCGCCCGTCACACCACGAGAGTTTGCAACACCCGAAGTCG
GTGAGGTAACCTTTATGGAGCCAGCCGCCGAAGGTGGGGCAGATGATTGGGGTGAAGTCGTAACAAGGTAGCC

>_isol_63
TGTTAGGGGGTTTCCGCCCCTTAGTGCTGCAGCTAACGCATTAAGCACTCCGCCTGGGGAGTACGACCGCAAGGTT
GAAACTCAAAGGAATTGACGGGGACCCGCACAAGCGGTGGAGCATGTGGTTTAATTCGAAGCAACGCGAAGAACCT
TACCAAATCTTGACATCCTCTGAYCCCTCTAGAGATAGAGTTTTCCCCTTCGGGGGACAGAGTGACAGGTGGTGCA
TGGTTGTCGTCAGCTCGTGTCGTGAGATGTTGGGTTAAGTCCCGCAACGAGCGCAACCCTTAAGCTTAGTTGCCAT
CATTAAGTTGGGCACTCTAAGTTGACTGCCGGTGACAAACCGGAGGAAGGTGGGGATGACGTCAAATCATCATGCC
CCTTATGATTTGGGCTACACACGTGCTACAATGGACAATACAAAGGGTAGCGAAACCGCGAGGTCAAGCAAATCCC
ATAAAGTTGTTCTCAGTTCGGATTGTAGTCTGCAACTCGACTATATGAAGCTGGAATCGCTAGTAATCGTAGATCA
GCATGCTACGGTGAATACGTTCCCGGGTCTTGTACACACCGCCCGTCACACCACGAGAGTTTGTAACACCCGAAGC
CGGTGGAGTAACCATTTGGAGCTAGCCGTCGAAGGTGGGACAAATGATTGGGGTGAAGTCGTAACAAGGTAACC

_ _

98 % identity

>_isol_52
GTTGTTCCCTTGAGGAGTGKCTTCCGGAGCTAACGCGTTAAGTCSACCGCCTGGGGAGTACGGCCGCAAGGTTAAA
ACTCAAATGAATTGACGGGGGCCCGCACAAGCGGTGGARCATGKGGTTTAATTCSATGCAACGCGAARAACCTTAC
CTGGTCTTGACATCCACAGAAMTTTCCRGAGATGGATTGGTGCCTTCGGGAACTGTGAGACARGTGCTGCATGGCT
GTCGTCAGCTCGTGTTGTGAAATGTTGGGTTAAGTCCCGCAACGAGCGCAACCCTTATCCTTTGTTGCCAGCGGTT
CGGCCGGGAACTCAAAGGAGACTGCCAGTGATAAACTGGAGGAAGGTGGGGATGACGTCAAGTCATCATGGCCCTT
ACRACCAGGGCTACACACGTGCTACAATGGSGCATACAAAGAGAAGCGAACTCKCGAGAGCAAGCGGACCTCATAA
AGTGCGTCKYASTCCGGATTGGAGTCTGCRACTCKACTCCATGAARTCGGAATCGCTAGTAATCGYAGATCASAAT
GCTACGGTGAATACGTTCCCGGGCCTTGTACACACCGCCCGTCACACCATGGGAGTGGGTTGCAAAASAAGTAGGT
AGCTTAACCTTCGGGAGGGCGCTTACCACTTTGTGATTCATGACTGGGGTGAAGTCGTAACAAGGGTAACC

_ _

96 % identity

>_isol_17
TGTMTAGTGGTTGGGGTTCCCCCCTTGTGCTGCCCTAACSCATTAARCACTCCCCCKGGGGAGTACGGGCSCAAGA
CTGAAACTCAAARGAATTGASGGGGGCCCSCACAAGCSGKGGAGCATGGGGTTTAATTCAAAACAACSCMAAAAAC
CTTACCRGGTCTTGATATCCTCTGACAACCCTAAAKATAGGGTTTTCCCTTCGGGGACAKAATGASRGGTGGTGGG
TGGTTGTCCTCCSCTCGTGTCGKGAGATGGTGGGTTAASTCCCRCMACCASCRCCCCCCCTTTATCTTTKTTGCCAW
TYTTTTKTTGGGCCCTCYAAGGTGACCGGCGGTGAAMAACCRGAAGAAGGGGGGGAAGACSTCAAATCCTCCYGCC
CCTTAAGACCTGGGCTACACMCGTGCTAMAATGGAAAAAAAAAAGGGCTGCAARACCSCGAGGTTTAACCCATCCC
AAAAATCTGTTCTCARTTCAGATCGCAATCCGCAACTCCACTGCGTGAAGCTGGAATCSCTAATAATCGCGGAACC
WCCTGCCGCGGTGAATTTYTCCCCGGGCCTTGTACACMCCCCCCCTCACACCACSAGARTTTGCAACCCCCSAAST
TSGTGAAGTCACTTTTRAGGMMCCCSCCCCCSAAGGGGGSGAARAATAWTGGGGTGAASTCATAACRRRKGKAAAC
C

>_isol_54
TGKRAGGTTGTGTTCYTKRAGRWGGCYTCCSGAACYWACSCSTTWAMTCCACCCCCCGGGGAKTTACGGCSCCARG
GTTAAACYCMAATGGATTTGMSGGGGCCCCCACAAACSGTGGAACATGGTGTTTAATTTCATGCCACCCSAAAAAA
CTTACCTACTCCTGACATCCAAAAAAATTTACAAAAAAGGTTTGGTGCCCTCSGGAACTCCKAAAAMRGTGCCGCM
TGGGTGTCCTCCMCTCCTGGTGKGAAATGGTGGGTTTAATCCCSCMACCARCSCCACCCTTATTCTTTTGTGCCCR
CSGTTCCGGCSGGAACTCCAARGAAAACGGCMRTGAAAAAMYGGAAGAAGGGGGGGAAGAACTCCARTCMTYCWGG
CCCTTAASAATAAGGCCACCCCCCTTCCACAATGGCATAAAAMAAAAAAAACCACCTCCCCARAACCARCCGACCT
CAAAAARTTTGTTSTAATCCSGAATGGAATCCGCCACTCCACTCCCWGAAATTCGAATCCCTTRTAATCCKAAAAT
ARAATTGCWCCGGTAATTWCTTTCCGGGSCTTGTTCMCCCCCCCCCTCCCMMCSGGGGAATGGGTTTCMAAARAAA
TTAGGTRCTTAACCCTCSGGGGGGCSRCTACCYMTTTTTTRTTTMTTGAYGGGGTGAAATTCMAAMMRGTWAAACC

_ _

86 - 80 % identity

>_isol_13
TGTMTAGTGGTTGGGGTTCCCCCCTTGTGCTGCCCTAACSCATTAARCACTCCCCCKGGGGAGTACGGGCSCAAGA
CTGAAACTCAAARGAATTGASGGGGGCCCSCACAAGCSGKGGAGCATGGGGTTTAATTCAAAACAACSCMAAAAAC
CTTACCRGGTCTTGATATCCTCTGACAACCCTAAAKATAGGGTTTTCCCTTCGGGGACAKAATGASRGGTGGTGGG
TGGTTGTCCTCCSCTCGTGTCGKGAGATGGTGGGTTAASTCCCRCMACCASCRCCCCCCCTTTATCTTTKTTGCCAW
TYTTTTKTTGGGCCCTCYAAGGTGACCGGCGGTGAAMAACCRGAAGAAGGGGGGGAAGACSTCAAATCCTCCYGCC
CCTTAAGACCTGGGCTACACMCGTGCTAMAATGGAAAAAAAAAAGGGCTGCAARACCSCGAGGTTTAACCCATCCC
AAAAATCTGTTCTCARTTCAGATCGCAATCCGCAACTCCACTGCGTGAAGCTGGAATCSCTAATAATCGCGGAACC
WCCTGCCGCGGTGAATTTYTCCCCGGGCCTTGTACACMCCCCCCCTCACACCACSAGARTTTGCAACCCCCSAAST

TSGTGAAGTCACTTTTRAGGMMCCCSCCCCCSAAGGGGGSGAARAATAWTGGGGTGAASTCATAACRRRKGKAAAC
C

>_isol_10
GTGTCTTGTGGTTGGGGTTCCCCCCCTTGTGCTMAKCTAACACATTAARCWCTCCCCCGGGGGAGTACGGTCSCAA
GACTGAAACTCAAAAGAATTGAGGGGGGCCCSCACAAGCYGKGGAGCATGGGGTTTAATTCAAAACAACSCRAAAA
ACCTTACCRKGTTTTGATCTCCTCTGACAACCCTAAAKATAGGTTTTTCTCTTCGGGGACAKAGTGACRGGTGGTG
GGTGGTTGTCCTCMSCTCGTGTCGKGAGATGGTGGGTTAASTCCCRCMACGASCRCCCCCCTTTATCTTTKTTGCC
AWTWTTTTKTTGGGCCCTCTAAGGTGACCGCCGGTGAAAAACCRGAAGAAGGGGGGGAAGACCTMAAATCMTCMTG
CCCCTTAAGACCTGGGCTACACACGTGCTAMAATGGAAAAAAAAAAGGGCTGCAASACCSCGAGGTTTAACCCATC
CCATAAATCTTTTCTTTKTTCAGATCGCAMTCTGCAACTCCACTGCGTGAAGCTGGAATCSCTAATAATCGCRGAT
CAWCMTGCCGCGGTGAATATYTTCCCGGGTCTTGTACACMCCCCCCSTCACACCAMGAKAKTTTGCAACCCCCRAA
STCKGTGAAGTCATCTTTATGGAMCCCSCCSCCRGAGGGGGGGCAGATTAKTGGGGTGAASTTMAAMMRWRGYACA
CAC

>_isol_61
CGTTGGATCCTTGAGACCCTATATGGCTCARCTTAASSMTTTARTTGATCGCCCGGGGAATAAGGRCCCMAGGWTT
GAACTCCAATGAATTGGASGGGGRCCCCMCCARCGGKGGAASMTGGTGKTTAATTTCAAACMACCSSRAAAAACTT
TACMRGSCTTGGAMTGCMTAKAACTTTCCTAAAAAGGATTGTTGCCTTCSGGAACTCTGACRCARGKGGTGGATGG
STGTCGTCMRCTCCTGGCGTGARAAGTTGGGKTAARTCCCCYAACSARCGCCACCCTTGTCCTTAWTTACCMSCAC
GTTATGGKGGGCACTCTAAGGARACTGCCGGGGACAAACCGGAGGAAGGGGGGGATGAMSYCAARYCMTCMTGGCC
CTTACRGCCTGGGCTACMCMCGTGCTACAATGGTCGGTACARAGGGTTGCCAASCCSCGAGGTGGARCTAATCTCA
CAAAACCGATCGTAGTCCGGATCGCAGTCTGCAACTCGACTGCGTGAAGTCGGAATCGCTAGTAATCGCGAATCAS
AATGTCSCGGTGAATACKTTCCCGGGCCTTGTACACMCCGCCCGTCACACCATGGAGAGTGKTGTTGCACCMGAAG
TAGCTAGTCTAACCTTCKGGRGSACGGTTACCAMRGKGKGATWCATGAYTGGGGTGAAGTCGTAACAAGGTAAACM

>_isol_01
ATGTCTAGAGGTTGGGGTTCCCCCTTGTGCTGCCCTAACRCATTAARCWCTCCCCCGGGGGATACGGGCAAAGACT
GAAACTCAAARGAATTGASGGGGGCCCSCACAAGCSGKGGAGCATGGGGTTTAATTCRAARCAACGCRAAAAACCT
TACCRGGTTTTGACATCCTCTGACAACCCTAAAKATAGGTTTTTCCCTTCGGGGACAKAATGACRGGTGGKGCGTG
GTTGTCCTCCSCTCGTGTCGKGAGATGGTGGGTTAAMTCCCRCAACSAGCSCCACCCTTTATCTTTKTTGCCARTW
TTTTKTTGGGCACTCTAAGGTGACCGCCGGTGAAMAACCRGAAGAAGGGGGGGAAGACSTCAAATCMTCMTGCCCC
TTATGACCTGGGCTACACACGTGCTAMAATGGAAAAAAAAAAGGGCTGCSACACCSCGAGGTTTAACCAATCCCAC
AAATCTTTTCTTAKTTCGGATCGCAMTCTGCCACTCCACTGCGTGAAGGTGGAATCSCTAATTATCGCGGAACCAC
CTGCCGCYGTGAATATYTTCCCGGGCCTTGTACACMCCCCCCGTCACACCACGAGAGTTTGCMACCCCCSAARTCK
GTGAAGTCACCTTTAAGGCMCCCSCCCCCSAAGGGGGGGCAGAATAATGGGGTGAASTCMTAAMGRRGTAAACC

>_isol_29
GTGTCTTGTGGTTGGGCCCCCKCCCTTGTGCTGAKCTAACACATTAARCWCTCCCCCTGGGGAGTACGGKCAAAGA
CTGAAACTCAAAAGAATTGGGGGGGGCCCRCACAAGCGGGGGAGCATGGGGTTTAATTCAAARCAACSCRAAAAAC
CTTACCRKGTTTTGATATCCTCTGACAACCCTAAAKATGGGTTTTTCTCTTCGGGGACAKAATGAGRGGTGGTGGG
TGGTTGTCCTCYSCTCGTGTCGKGAGATGGTGGGTTTACTCCCRCMACCASCRCCCCCCTTTATCTTTKTTGCCAW
TWTTTTKTTGGGCCCTCTGAGGKGACCGCCGGTGAAAAACCRGAAGAAGGGGGGGAAGACSTCAAATCATCRTGCC
CCTTAAGACCTGGGCTACACACGTGCTACAATGGAAAAAAAAAAGGGCTGCAACACCSCGAGGTTTAACCCATCCC
AAAAATCTGTTCTTTSTTCAGATCGCAMTCTGCMACTCCACTGCGTGAAGCTGGAATCSCTAATAATCGCRGATCA
WCRTGCCGCGGATAATTTYTTCCCGGGTCTTGTACCCCCCCCCCSTCACACCAMGAGAGTTTGCAACACCCSAAST
CGGTGAAGTMATCTTTRAGGAMCCCSCCSCCSAAGGKGGRGAAKATTATTGGGGTGAASTCAAARRKKGGYAAACA

>_isol_48
ATATCKATTGTAGTTGGTTCTTGGRGATGGCTTCCSGAATAACGCSTTAAMTCCACCCCCTGGGGAGTACSGCCSC
MAGGGTAAAACTCAAATGGATTGGSGGGGGCCCGCMCMAGCGGGGGAGCATGGGGTTTAATTCCATGCAACSCSAA
AAACCTTACCTACTCTTGACMTCCAAAAAACTTAACAAAAATGCTTTGGTGCCTTCSGGAACTCTGAAACMGGTGC
TGCRTGGGTGTCCTCMSCTCGTGTTGWGAAATGGTGGGTTAAMTCCCSCMACCARCSCCACCCTTATTCTTTTTTG
CCMGCSGTTCCGGCGGGAACTCMRARGARACCGGCMGTGAAAAACTGGAAGAAGGGGGGGAAGACCTCMARTCATC
MTGGCCCTTAASAATARGGCTACCCMCGTGCTACMATGGCATATAAMAARAAAAACCACCTCSCGAGAACCAGCCG
ACCTCAWAAARTATGTCMTAATCCSGATTGGAATCCGCCACTCCACTCCATGAARTCMGAATCSCTAATAATCSTA
WATCAAAATGCCACSGTGAAWTCCTTCCGCGGCCTTGKACACMCCCCCCCTCACCMCRTGGGAGTGGGTTGCAAAA

RAARTTAGTAKCTTYAYCCTCRGGAGGGCSCTTACCWYTTTGTTAATYAWGASTGGGGWGAASTCCAAMSARGKMA
AACA

>_isol_51
ATAAGATTGTGYGTTTGCCTTTTAGCGTGGCTTCKGAATACGCGTTAACTCCACCCCCTGGGGAGTACCGCCSCMA
GGTTAAAACTCAAATTAATTGACGGGGGCCCRCACMARCGGTGGAGCATGGGGTTTAATTCKATGCAACGCGAAAA
ACCTTACCTACTCTTGACATCCAAAAAATTTTCCAAAAATGGATTGGTGCCTTCRGGAACTCTGAAASAGGTGCTG
GGTGGGTGTCCTCWSCTCGTGTTGWGAAATGGTGGGTTAACTCCCRCAACSAGCRCCCCCCTTATTCTTTGTTGCC
MGCKGTTACGCCGGGAACTCAAAAGAAACCGCCWGTGAAAAACTGGAAGAAGGGGGGGAAGACCTCWARTCATCRT
GGCCCTTAAKAATAGGGCTACCCMCGTGCTACAATGGCSCATAAAAAAAAAAACCACCTCSCGAGAACAAGCRGAC
CTCAAAAAGTGCCTCRTAMTCCRGATTGGARTCCGCCACTCCACTCCSAGAASTCAGAATCSCTAATAATCSTAMA
TCAAAATGCCRCGGTKAATACCTCCCCGGGTCTTGTACACCCCCCCCSTCACACCRTGGAGTGTGTTRCAAAARA
ARTTAGTWTCTTCYTCTTCMGGAGGGCKCTTACCTTTTTGTTATTCAAGASTGGGGWGAASTCWAAARRGGKAAAC
A

>_isol_25
CKTGTYTAGTGGTTGGGGTTCCCCCCCTTGTGCTGAKCTAACRCATTAARCWCTCCCCCTGGGGAGTACSGWCSCA
AGAGTGAAACTCAAAAGAATTGAGGGGGGCCCRCACAAGCYGTGGAGCATGGGGTTTAATTCRAASCAACGCRAAA
AACCTTACCARGTCTTGACATCCTTTGACCACCCTAAAKATAGGGTTTTCCCCTTGGGGACAAAAWGAGAGGTGGT
GSGTGGTTGTCCTCCCCTCKTGTCGYGAGATGTTGGGTTAACCCCCCCCACSAGCSCCCCCCTTTTTCTTTTTTGC
CCCCTTTTTTTTGGGCCCTCTAAKGTGACCGCCGGTGACAAACCRGAAAAAGGGGGGAAAACMACAAATCATCWT
GCCCCTTATKATCTGGGCTACACACGTGCTACAATGGAAAAAAAAAAGGGGTGCAAMACCCCGAGGTTTAACCCAT
CCCAAAAATTTTTTCTCTTTTTTGATAGTAKTCTGCAACTCCACTGCGTGAAACTGGAATCCCTAATAATCGCAGA
ACAMCRTGCCGCGGTGAATATTTTCCCGGGTSTTGTACACACCCCCCCTCACACCACRAGAKRTTTCAACACCCCA
ASWCGGKGARATAACCTTTATRGARCCCCCCCCCSAARGTGGGGAAAAATATTGGGGKGGAGTYCTMACRRRGKSM
AAC

>_isol_43
TGTTTAATGTTTAGGGTTCCCCCTTTKTGSTKAATTACSCATTAARCACTCCCCCTGGGGAGTACSGGCSCAAGGG
TGAAACTCAAAAGAATTTAMGGGGGCCCSCACAARCSGTGGAACATGTGGTTTAATTTCAAACAACSCSAAAAACC
TTACCARGTCYTGACATCCTCYGAAAACCCYAAAAATARGGCTTCCCCCTCSGGAACARAATGACMGGTGGTGCAT
GGTTGTCCTCCSCTCSTGTCSTGARATGTTGGGTTAAMTCCCSCAACCARCSCCACCCTTTATCTTTRTTGCCATC
AATTARTTGGGCACTCYAARGTGACCGCCGGTGAAMAAMCSGAAGAAGGGGGGGAAGACCTCMAATCATCMTGCCC
CTTAAGACCTGGGCTACACACSTGCTACAATGGACSGTAAAAARAGCTGCAAMACCSCGAGGTGGAACTAATCCCA
AAAAACCSTTCYCARTTYMGAATGGAAGCCGCCACCCCCCTACMTGRARCTGGRATCCCTARTAATCCCSGATTCR
CCTGCCGCCGTKAATACCTTCCCCGGCCYTGKACCMCCGSCCSTCMCACACGARWGTKTTGKAACCCCSAAGTYSG
TKGGGTWACTTTTTAGGACCMRCCYCTKAGTGGGAMAGATGATGGGGTGAMTCSTAMGAGGTAACCAAATTAA

>_isol_59
GSTCKTTGTGYGTGTGCCCTTGSGGTGGCTTCKGAATACGCKTTAACTCCACCCCCTGGGGAGTCGGCCGCWGGGT
TAACACTCAAATTAATTGAGGGGCGCCCRCACAAGCYGTGGAGCATGGGGTTTAATTCTATGCAACGCAGAAAACC
TTACCCACTTTTGATATCCAAAAAATTTTYCAAAAATGGTTTGGTGTCTTCRGGAACTCTGAAAGRGGTGCTGCGT
GGSTGTCCTCMYCTCGTGTTGAAAAATGGTGGGTTTACTCCCRCMAMSASCRCCCCCCTTCTTTTTTTTTGCCCGC
TGTTACGCCGGGAACTCARAAGAWACCGCCWGTGAAAAACTGGAAGAAGGGGGGGAAGACCTCWASTCMTCGKGGC
CCTTAAKAATAGGGCTACCCMCGTTCTACWATGGCSCAWAAAAARAAAAACCACCTCSCGAGAACCAGCRGACCTC
AAAAAGTGCGTCRTAMTCCRGATTGGASTCCGCCACTCCACTCCATGAASTCAGAATCSCTAATWATCRWAYATCA
WAATGCYRCKGAKAATTTCTTCCCSGGTCTTGTACACMCCCCCCSTCACAMCRTGGGAGTGTGTTGCAAAAGAART
TAGTWTCTTCATCTTSRGGAGGGCTCTTACCTCTTTGTGATTCAWGAGTGGGGTGRASTCAYAACGGKRGYCACAC
C

>_isol_71
GGTTYAAGTTGRTTTGGGGGCTTCKGACTACGCGTTAATWACCCCCTGGGGATCCGCCGCKGGTTAACACTCAAAT
GAATTGACGGGCGCCCRCACAAGCGGTGGAGCATGGGGTTTAATTCTATGCAACGCRAAAAACCTTACCTACTCTT
GACATCCARAAAATTTTCCAAAKATTGATTGGTGTCTTCRGGAACTCTGASASRGGTGCTGCGTGGSTGTCCTCYY
CTCGTGTTGTAAAATGTTGGGTTAACTCCCRCMACSAGCRCCCCCCTTATTCTTTGTTGCCMGCTGTTACGGCGGG
AACTCAAAAGAKACCGCCWGTGAAAAACTGGAAGAAGGGGGGGAAGACCTCYARTCATCRTGGCCCTTAAKAATAG
GGCTACCCMCGTGCTACAATGGCSCAAAAAAAAAAAAAACCACCTCSCGAGAACCAGCRGACCTCAAAAAGTGCGTC
MTAMTCCRGATTGGARTCCGCCACTCCACTCCAAGAASTCAGAATCSCTAATAATCSWAMATCAWAATGCCRCSGA

KAATATCTTCCCSGGCCTTGTACACCCCCCCCSTCACAMCRKGGGAGTGTGTTGCAAAARAARTRWGTMTCTTAAC
TTTYRGGAGGGCKCTTACCWTTTTGTTYMTCAATGMTGGGGAGAASTCMMAAMRRWKGKAAACAC

>_isol_08
TGTYTTGTGGTTGGGGTTCCKCCCTTGTGCTGCCCTAACGCATTAARCACTCCCCCTGGGGAGTACGGTCSCAAGA
CTGAAACTCAAARGAATTGRSGGGGGCCCSCACAAGCGGGGGAGCRTGKGGTTTAATTYCAAACAACSCSAAAAAC
CTTAMCRRGTCTTGACATCCTCTGACAACCCTAAARATARGGCTTTCCCTTCGGGGACARAATGACMGGGGGTGCA
TGGTTGTCCTCCSCTCGTGTCGKGAGATGGTGGGTTAASTCCCSCMACCAGCGCCACCCTTGATCTTTRTTGCCCR
CCTTTTKTTGGGCACTCYAAGGTGACCGCCGGTGAAMAACCSGAAGAAGGGGGGGAAGACCTCMAATCMTCMTGCC
CCTTAAGACCTGGGCTACMCMCGTGCTAMAATGGAAAAAAAAAAAGGGCTGCMARACCSCRAGGTTTAACCMATCCC
AAAAATCTGTTCTCARTTCRGAACSCAATCCGCMACTCCACTGGSTGAARCTGGAATCSCTAATAATCSCSGAACA
ACCTGCCGCGGTKAATAWCTTCCCSGGCCTTGTACACMCCCCCCSTCMCACCAMSAGARTTTGCMACCCCCSAAST
CSGTGAAGTAACCTTTATGGAMCCCSCCSCCSGARGGGGGGCAGAATATTGGGGTGRASTCMTAAGMRGGYAAACC

>_isol_24
GTCTAGAGGTAGGCCCTCCCTTGTGCTCCCTAACACAATAARCCTCCCCCTGGGGAGTACSGTCAAAGASTGAAAC
TCAAAAGAATTGACGGGGGCCCSCACAAGCYGKGGAGCATGGGGTTTAATTCRAARCAACSCSAAAAACCTTACCR
GGTCTTGACCTCCTCYKAACCCCCCAAAAAKKGGGTTTTCCCTTCGGGGAAAKGGTGASRGGTGGKGGGTGGTTGT
CCTCMSCTCGTGTCGKGAGATGGTGGGTTAASTCCCRCMACSAGCRCCACCCTTTTTTTTTKTTGCCAWTWTTTTK
TTGGGCCCTCTAAAGAGACCGCCGGWSAAAAAACRGAAGAAGGGGGGGAAGACSTCAAATCATCCTGCCCCTTAAG
ACCTGGGCTACACMCGTGCTACAATGGAMAGAACAAAGGGCTGCCASACCGCGAGGTTTARCCCATCCCATAAAAC
TGTTCTCAKTTCAGATTGCARGCTGCMACTCCGCTGGMTGAARGTAGAATCSCTAATAATCGCRGAACAWCMTGCC
GCGGTGAATTTYTTCCCGGGSCTTGTACACMCCCCCCSTCACACCAMGAGAKTTTGCAACCCCCSAASTCSGTGAA
GTAACCTTTAWGMAMCCCCCCCCCRAAGGGGGGGAAAATTATTGGGGTGAATTCMAAMRRRGGRAAAcc

>_isol_27
CCTGTGTTAGGRGTTTCCKCCACYTWATGCYKCCCTAACSCATTWAACACTCCCCCCGGGGAGTACSGTCSCAAGA
MTGAAACTCAAARGAATTGRMGGGGGCCCSCMCAARCSGTGGARCMTGTTGTTTAATTYCAAACMACSCSAAAAAC
CYTACCARGTCYTGACMTCCTCYGACAACCCYAAARATARGGCTTTCCCTTCGGGGACARAATGACMGGTGGTGCA
TGGTTGTCCTCCSCTCSTGTCSTGARATGTTGGGTTAAMTCCCSCMACCARCSCCACCCTTTATCTTTWTTGCCAR
CMTTTTRTTGGGCCCTCYAARGTGACCGCCGGTGAAMAAMCSGAAGAAGGGGGGGAAGACCTCMAATCATCMTGCC
CCTTAAGACCYGGGCYACCCMCSTGCYAMAATGGAAAAAAAAAARGGCTGCCAAACCSCMAKGTTTAACCCATCCC
AAAAATCTGTTCTCAWTTCSGAACSCAATCCGCAACTCCACTGCSTGAARCTGGAATCSCTAATAATCCCSGAACA
ACCTGCCSCGGTGAATACCTTCCCGGGCCTTGTACACMCCSCCCSTCACACCAMSARARTTTGCMACMCCCSAART
YSGTGAAGTAAYCTTTAWGGMACCCRCCSCCSAARGKGGGGSARAWKRWTGGGGTKAARTYCTAACRAAGKAACAC
C

>_isol_46
AAAAMGCTTATGTTGGRKTTTAMTTTCTCTGTATCSARCTAAACAATAARCACTCCCCCKGGGGAATAACACCGCM
AGGGTKACYCTCMAAAGAATTGGMGGGGGCCCCCACAAACCGGGGAACATGTGGTTTAATTTCAAACAACCCSAAA
AACTTTAGCGGTTTTGGAATACTCTGMGAATCCAAAAGAAAAAAGATCTTTCCTCACGAAAAAAAAAAAGGGGGGGG
GTGTTTGTTCCCACCCCKCGTGGAAGGGTTTGTTTAATCCCCCCCCCCCCCCCACCCCCCCTTTTTTTTTTTCCCCC
CCCTTTTTGGGGGGCKCCCAAAAAAAAAACCCGCGGGAAAAAMMRGAAAAGGGGGGGGGGGGRKAWAAYYTYYYSCC
CCCTTTTCCCCTGGGGGACRCGGGGTKCACAAGGGGGGGGGAAAGGAGGTACCAAAAASCSMKAAATAAAGKTTTT
TTTAAAAAACTCTTTAAAATTCTTTTYTKKGGAGGAAAGMCCGCCCRCAACYCCCCCTTAATGAAAKSAAGAAKCS
MAAAATAACCCCGGGATTAAAACCCCCCGGGGAATTTTTTWCCGGGGSTTTTTAACCMCCSSKTTTTTYWWCCARG
GGGAGTTGGGGAKTCCCCCAAAWTKGGKGGCCCCMCYCSYRAAGRASSCCCCCCAAAAAAAAAAAAAMYYATARGG

>_isol_58
AAKKMTKRTGTGTTTGGGTTTTATCCCTTTGTGCCRAAATTCACYTTAARCWTTCCCCCGGGGGAGTACGGTCSCA
AGACTGAAACTCRAAAGAATTGAGGGGGACCCSCACAARCWGTGGAGTATGGGGTTTAATTCAAAACAACSCRAAA
AACCTTACCRKGTTTTGATATCCYTATGACCSKTMAAAAAATGKTTTTTTTCTTCRGGACAWAAAARASRGGTGGT
GGGTGGTTGTCCTCCSCTCGTGTCGKGAGATGGTGGGTTAACTCCCRCMACSASCRCCCCCCTTTATCTTTKTTGC
CAMCTTTTYKGGGGGGCCCTCYRAGGTGACCGCCGGTGAAMAACCGGAAGAAGGGGGGGAAGACCTCAAATCMTCC
CGCCCCTTAAGACCTGGGCTACACMCSTACTACAATGGCCGGWACAACGGGGTGTGAASCCSCGAGGTGGAAASAA
TCCTAAAAASCCSGTCTCARTTCSGATTGGCSGCTGCCACTCCCCTGCAAGAASTCAGAATTGCTAATAATCSCKG
AACAWCCTGCCGCGGAGAATATYTTCCCGGGTCTTGKACACMCCCCCCSTCMCACCAMGAKTTTTTATAACCCCCR

AYSTCGGGGGGATAACCACAARGAMCCCSCCSCAMAARGGGGRAAARATTAKTGGGGAGAASTCAAAMRRARTAAC
ACC

>_isol_69
TGTYAAGTGGTTGGGCCCCCCCCTTGTGCTGAKCTAACACATTAARCCTCCGCGGGGGGAAACGACCGGRGGGGTG
AAACTCAAAAGAATTGGGGGGGACCCRCACAAGCGGGGGAGCATGGGGTTTAATTCAAARCAACGCRAAAAACCTT
ACCAAATCTTGATCTTTTTTGACCSCTCTAAAKATAKAKTCCTCCCCCTCGGGGGACAAAGTGACRGGTGGYGCGT
GGTTGTCCTCCSCTCGTGTCGKGAGATGGTGGGTTAACTCCCMCMACSAGCRCCCCCCTTAATCTTTKTTGCCATT
AATTTKTTGGGCCCTCTTTKTTTACCGGCGGTGAAMAASCRGAAGAAGGGGGGGAAGACCWCAAATCATCCCGCCC
CTTAATATTTGGGCTACACMCGTGCTACWATGGAAAAAAAAAAGGGCAACTAAACCGCGAGGTCCAACAAATCCCA
AAAAGTTTTTCTTTKTTCATATTGTAMTCCGCMACTCCAACACSAGAAGGTGGAATCSCTAATAATCMTAMAACAW
CRTGCTACGGAGAATATYTTCCCGGGTCTTGWACACMCCCCCCGTCACACCAMGAWTTTTTGTAACCCCASAASCC
GGTGGAATCACTTTTTRWGGAKCTCSCCCTASGAGGGGGGAAARATGAKTGGGGWGAASTCAMAARRRRGTCACA
C

>_isol_33
CGTCTCATGTGTTTKAGSCCCCCCTTTTGTGGTGAKCTAACACATTAARCWCTCCCCCTGGGGAGTACGGTCMCKG
GGTGACTCTCAAAAGAATTGAGGGGGACCCRCACAAGCGGKGGAGCATGGGGTTTAATTYAAAACAACSCRAAAAA
CCTTACCAACTTTTGATATCCCCCTGACCGGTACAAAAATGTACCCTCCTCTTCGGGGGCGGGGGTGACRGGTGGT
GCGTTGTTGTCCTCMYCTCGTGTCGKGAGATGGTGGGTTAACTCCCRCMACSAGCSCCCCCCTTGTTCTTTKTTGC
CAYTWTTTTKTTGGGCACTCTRARGARACCGGCGGTGAAAAAMCRGAAGAAGGGGGGGAAGACCTCAAATCATCRT
GCCCCTTAAGAGTTGGGCTACACMYGTTCTAMWATGGARRGKAAAAAGGGCARCMAAMCCSCGAGGTGGAACCCAT
CCCAAAAAGCCSTTCTTTSTTCAGATTGGSRGCTGCCACTCCCCTGGAAGAARTCAGAATCSCTTATAATCSGRKG
TCAWCATACTGGGGAKAATTTSTTCCCGGGTTTTGTACACMCCCCCCSTCACACCAMGAKATTTTGTAACCCCCSA
ASTCKGTGAKGTCACCTTKAGGCSCCCSCCSCASAAGGGWGGAMAKATTAGTGGGGTGAASTCAMAAGGKWGKYAA
CC

>_isol_44
KTGTYTTGTGGTTGGTGTCCCCCCCCTTGTGcTGAtCTAACACaTTAAaCaCTCCCCCTGGGGAGTACGGTCAaAGA
STGAAACTCAAAAGAATTGAGGGGGGCCCaCACAAGCgGGGGAGCRTGGGGTTTAATTCAAAACAACGCaGAAAAC
CTTACCGgGTTTTGACCTCCTCTGACAATCCtAAAaATAAGACCCCCCCCTCGGGGGCAgAGTGAGgGGGGGtGGG
TGGTTGTCCTCCYCTCGTGTCGtGAGATGGTGGGTTAACTCCCaCMACCAGCSCCCCCCTTGaTCTTTKTTGCCAa
TYTTTTKTTGGGCWCTCTAAGGTGACCGGCGGTGAAAAACCRGAAGAAGGGGGGGAAGACCTMAAATCCTCcTGCC
CCTTAASACCTGGGCTACACACGTGCTAcWATGGAAAAAAAAAAAGGGCARCAAAACCgCGATGTTAAaCCCATCCC
AAAAATCTTTTCTTTTTTCAGATCGcAYTCTGCAACTcCACTGCGTGAAGGTGGAATCSCTAATTATCGCAGATCA
aCRTGCCGCGGATAATATYTTCCCGGGTCTTGTACACMCCCCCCSTCACACCAMGAGATTTTGTAACCCCASAAST
CGGKGAKGTCACCTTTRARGAMCCCSCCSCCSAAGGGGMGAAAKAATAKTGGGGTGRASTCATAAGGRKGKMCAAC

>_isol_64
TGTCTAGAGGTTGGWTCCCKCTTTGTGTGAKCTAACACATTAAMWCTCCCCGGGGGGAGTCCACCGCGGGGTGAAA
CTCAAAAGAATTGAGGGGGACCCRCACAAGCKGTGGAGCATGGGGTTTAATTCRAAACAACSCRAAAAACCTTACC
AAATCTTGATCTTCTTTRAAAACTCWAAARATARAWTCCTCCCCCTCGGGGGACAAAATGACRGGTGGYGCGTGGT
TGTCCTCCSCTCGTGTCGWGAGATGGTGGGTTAACTCCCRCAACGAGCRCCCCCCTTAATCTTTWTTGCCATTAAT
TTKTTGGGCCCTCTTTGTTTGCCGCCGGTGACMAACCRGAAGAAGGGGGGGAAGACCTCAAATCATCCCGCCCCTT
AATATTTGGGCTACACACGTGCTACMATGGAMAATAAAAAGGGCCAATAAACCGCGGGGTCRTGCAAATCCCAAAA
AGTTTTTCTCTKTTCAGATTGTASTCCGCCACTCCAACACAAGAAGGTGGAATCSCTAATAATCRTAMAACAWCRT
GCCACGGAGAATATYTTCCCGGGTCTTGKACACMCCCCCCGTCACACCAMRAKATTTTGTAACACCCRMCGGCGGW
GGAATCACTTTTTRWGGWWCTWGCCSTARGAGGGGGGAAAAATTAKTGGGGGTGAASTCAWAACGRRGTCAACC

>_isol_68
TGTTAGRGGGTTTCCGCCCYTTAGTGCTGMAGYTAACGCATTAAGCACTCCGCCTGGGGAGTACGRCCGCAAGGYT
GAAACTCAAAGGAATTGACGGGGRCCCGCACAAGCGGTGGAGCATGTGGTTTAATTCGAAGCAACGCGAAGAACCT
TACCARRTCTTGACATCCTYTGACCGCTCTAGAGATAGAGYYTTCCCCTTCGGRGSACAAAGTGACRGGKGGYGYA
TGKKTGTCSTCMKCTCGTGTCGWGAGATGTTGGKTTAASTCCCGCAACGAGCGCACCCCTTAATCTTATTTGCCAT
CWTTAWKTTGGRCACTCTAAKGTGAGTGCCGGWGACAAACCGGAAGAAGGKGGRKATGACSTCAAATCMTCRTGCC
CCTTATGATTTGSKMTACACRCGTGCTACWGTGGACAATACAAAGGGSMGCTAAACCGCGAGGTCRAGCAAATCCC
ATAAAGTTGTTCTCAKYTCGKATTGTASTCTGCMWCTCSACTACRTGAAKCTGGAATCGCTAGTAATCGTAKATCA
RYATGCTACGGWGAATACGTTCCCGGSTCTTGTACACACCGCCCGTCACACCACGAGAGTTTGTAACACCCGAASC

CGGTGGAGTAACCWTTTTATGGMKCTAMCCCSTCGAAGGTGRSACAAATGATTGGGGWGAASTCRTAACRARGGTA
ACC

>_isol_12
AACAGTTTGGGTTCCTCCCTTGYGCTCCCCAAYATTWWMCCTCCSSGGGGGGAAACGGAAGAAAGASAGAAACTCA
AAAGAATTGAGGGGGGCCCACACAAGCYGGGGAGCATGGGGTTTAATTCRAAACAACSCRAAAAACCTTACCRKGT
CTTGACCTCCTCTGACAACCCTAAARATGGGGTTTTCCCCTCGGGGACARAATGACRGGTGGTGCGTGGTTGTCCT
CCCCTCGTGTCGKGAGATGGTGGGTTTACTCCCACMACCAGCACCCCCCTTTWTTMTTKTTGCCAWTWTTTTKTTG
GGCCCTCTAAGGTGACCGCCGGTGAAMCACCSGAAGGAGGGGGGGATGACCTAAAATCCTCCCGCCCCTTAACACC
TGGGCCACACACGTTCCAAAGTGGAAAAAAAAAAGGGCTGCAACACCRCGGGGTTTCACCCATCCCATTAATTTTT
TWTTTTTTCAGAACGYAYTCCGCAACTCCAGCGCGTGAAGGSGGCATCKCTAATAATCGCSGATCAACCCGCCGCG
GAGAATACCTTCCCGGGCCTTGTACACMCCCCCCGTCACACCAAGAGAGTTTGCCCCCRCAMAACGCGGGGAKGTA
ATCTTTAAGGACCCCGGCGCCGAAGGTTGGGAKATGGTRTTTGGTGKACTCYYACRAGTCCACC

>_isol_30
NNNNN

>_isol_42
SGSGAAGATTTWGGTCCTCCTTTGTGCWCCCTTCACTTYCCTCGCGGGGGGATCGCTCARAGASAGAACTAAAAGT
TTYAGGGCGCCCRCAAACYGTAGAGCATGGGGTTTTTTCAAACAACGCRAAAACCTTACCGKGTCTTGATCTCCTC
TGACWATCCWAAAKAKAAGACCCCCCCCTCGGGGGAAKAATGAGRGGTGGYGGGTGGTTGTCCKCCSCTCGTGTCG
KGAGATGGGGGGTTTACTCCCRCMACSAGCRCCCCCCTTTATTTTTTTTGCCAWTTTTTTTTTGGGCACTCTGAGG
TGACCGGCGGTGAAAAACCRGAAGAAGGGGGGGAAGACCTMAAATCATYCCGCCCCTTAASACCTGGGCTACACAC
GTGCTACWATGGAAARAAAAAAGGGCAGCRACACCGCGATGTTAARCCWATCCCACAAATCTGTTCTCTSTTCAGA
TCGCASTCMGCWACTCCACTGCGAGSAGCTAGWATCKCTAATWATCGCKGATCAWCRTGSCGCGGAGAATATKTTC
GGGSGTCTTGTACACACCSCCCGTCACCACCACGAGAKTTTRTAACMCCMGWCKKTGAGGWWRTCAYCTTTTGWAG
MRSCMGCCGMAGATGKKRCGACATGATTARTGGKGWGWAGTMYAAMGRWWRTCAACCAACTWAG

>_isol_55
AGTGTCTAGTGGTTGGGTTCCKCCCCTTGTGCTKCCCTACACATTAARCACTCCCCCGGGGGAAACGGGCSCAAGA
CTGAAACTCAAAAGAATTGACGGGGGCCCSCACAARCSGTGGAACATGGGGTTTAATTCAAAACAACGCSAAAAAC
CTTACCRRGTCTTGACATCCCSTTGACCACTGKAAAAATATTTTTTCCCCTTCGGGGGCAAAKGWGACGGGGGGYG
CGTGGTTCTCCTCCYCTCGTGTCGAGAGATGGTGGGTTAACCCCCRCAAACAGCSCCCCCTTTTATTTTTTTTCCC
ATTTTTTTTTTGGGCACTCWAGGGGGACCGCCGGAGACAAACCAGAAGAAGGGGGGGAAGACCTCAAATCCTCCTG
CCCCTTAAGACCGGGGCTACACMCGTTCTAAAAGGGAAAAAACAAAMGGGTGCCAACTCGCGAGAGGGAACTAATC
CAAAAAARTCTTTCTCARTTCGGATTGGARGCTGCCACTCCCCTACSTGAAACCSGAATCSCTAATAATCCCGGAA
CAWCCTGCCGCGGATAATTTCTTCCCGGGTCTTGTACACACCCCCCSTCACACCAAAATTTTTTAYCACACCASWA
STCGGWGAAGTCACTTTTTGGAACCCCCCCCCGAAGGGGGAAAAWAAATAGTGGGGAGAASTTAYAAMGRRAAAAC
ACC

_ _

79 - 70 % identity

>_isol_02
YTAGAGTGTTAGGGGTTTCCKCCWCYTAGTGSTKCAGCYAACGCATTAAACACTCCCCCTGGGGAATACSGTCSCA
AGGSTGAAACTCAAARGAATTGACGGGGGCCCRCACAARCCGTGGAACATGTGGTTTAATTTRAARCAACSCSAAA
AACCTTACCAAGGCTTGACATCCTTTGACAACCCTAAAAATAGGGCTTTCCCCTTGGGGGCAAAATGACAGGTGGT
GCATGGTTGTCCTCCCCTCKTGTCGTGAGATGTTGGGTTTAATCCCCCCACCASCSCCCCCCCTTTTCTTTTTTTC
CCCCYTTTTTTTTTGGCCCCCTAAWGTGACYGCCGGTGACAAAACRGAAGAAGGGGGGGAAGACMWCAAAACATCAY
GCCCCCTATAAMCTGGGCKACACACSTGCTACAAWGGAAAAAAAAAARGGGGGCMAMAACCCGRGGTTTAACCCAW
CCCACAAATTTTTTCTCYKTTTTGAAAGTARTCTGCSACTCCACTGCSTGAAACTGGAAACCCTAATAATCGCCGA
ACCACATGCCGCCGTGAAAAATTTCTCCGGCCTTGTACACACCCCCCCTCAAAACACAAGAGTRTGCAACACCCCA
AATCCGTGGAGTAACWCTTTTTTGAACCCCCCCCCCMAAGGGGGGGRAAAARAWTGGGGGGAARTCCTWAMAAAGK
WAACAA

>_isol_60
AAAATATMMGWAGTTTGCCTTTTAGCGTGGCTTCCKGAATACGCGTTAASTCCACCGCCTGGGGAGTACGGCCGCM
GGGTAAAACTCAAATGAATTGACGGGCGCCCSCACMARCSGTGGAGCATGGGGTTTAATTCYATGCAACSCSGAAA
ACCTTACCCACTCTTGACATCCARAAAATTTTCCAAAGGTGGGTTGGTGCCTTCRGGAACTCTGAAASRGGTGCTG
GGTGGGTGTCCTCCSCTCGTGTTGWRAAATGGTGGGTTAASTCCCRCMACCAGCSCCACCCTTATTCTTTGTTGCC
MGCKGTTAMGGCGGGAACTCAAAAGAAACCGCCMGTGAAAAACTGGAAGAAGGGGGGGAAGACCTCMASTCCTCMT
GGCCCTTAASAATAGGGCTACCCMCGTGCTACAATGGCSCATAAAAARAAAAACCACCTCCCGAGAACCAGCGGAC
CTCAAAAAGTGCGTCMTAATCCSGATTGGAATCCGCCACTCCACCCCSAGAARTCRGAATCCCTAATAATCSKAKA
ACAAAATGCCACSGAGAATACCTTCCCGGGCCTTGTACACCCCCCCCCTCMCACCRTGGGAGTGGGTTGMAAAARA
ARTTAGTAKCMTAACCCTCRAGGAGGCYCTTACCWYTTTGTTAATCAAGAMTGGGGTGAAMTCATAAASRRGTMAC
C

>_isol_66
GTGTCTTGTGGTTGGGCCCCCCCCCKCTGTGCTGAKCMAACACATTAASCACTCCCCCGGGGGAGTAACACCGCWK
GGGTGACACTCAAAAGAATTGAGGGGGACCCRCACAAGCGGGGGAGCATGGGGTTTAATTCAAARCAACGCRAAAA
ACCTTACCAAATTTTGATCTTTTTTGACCSCTCTAAAKATAKAKTTCTCCCCCTCGGGGGACAAAGYGACRGGGGG
YGGGTGGTTGTCCTCCCCTCGTGTCGKGAGATGGTGGGATAACTCCCMCMACSAGCRCCCCCCTTAATCTTTKTTG
CCATTAATTTKTTGGGCCCTCTTTTTTTACCGGCGGTGAAAAACCRGAAGAAGGGGGGGAAGACCWMAAATCCTCC
CGCCCCTTATTATTTGGGCTACACMCGTTCTAMWATGGAWAATAAAAAGGGCAKCTAAACCGCGGGGTCCAACAAA
TCCCAAAATGTTTTTCTCTTTTCATATTGTAMTCCGCMACTCCACTACAAGAAGCTGGAATCYCTAATAATCMWAM
AACAWCRTGCTACGGTKAATTTYTTCCCGGGTCTTGKACCCCCCCCCCSTCACACCAMGAKATTTTGYAACCCCCS
AASCCGGWGGAATCACTTTTTGAGGAACTCGCCCTCSGAGGGGGGAAAWATGWGTGGGGWGAASTCMAAMRRKRGY
CC

>_isol_70
AGYTAAGGTTGGGGTTCCCCCYTKTGCTGCCCTACSCATTAARCACTCCCCCYGGGGAATACSAACGCMAGGGTGA
AACTCAAARGAATTGASGGGGACCCSCACAARCSGTGGAACATGGGGTTTAATTTCAAACAACSCSAAAAACCTTA
CCAAATCYTGACATCCTTTGACCSCTCTAAAAATARARTCCTCCCCCTCGGGGGACAAARTGACSGGGGGKGCRTG
GTTGTCCTCCSCTCGTGTCSWGAAATGGTGGGTTTAMTCCCRCAACCARCSCCCCCCTTAATCTTTWTTGCCCTTA
TTTAKTTGGGCACTCYAARTTTACCGGCGGTGACAAAMCRGAAGAAGGGGGGGAAGACCTCAAATCCWCCYGCCCC
TTAATATTTGGGCCACACMCGTTCTAAAATGGAAAAAAAAAAAGGGCCRCTAAACCGCGGGGTCAAACAAATCCCAA
AAAGTTTTTCYCMWTTYRGAATGGAMTCCGCCACTCCACCACCTGAARCTGGAATCCCTAATAATCCKAAAACARC
CTGCCWCGGTGAATACCTTCCCGGGTCTTGGACMCCCCCCCCCTCACACCAMGARAKTTTGWAACCCCCSAAMCCG
GTGGAATAACCYTTTAWGGAACCCMCCCTCCAARGGGGGAAAAATTAKTGGGGRGAAMTCMTAACSAAGTAACC

>_isol_72
GTYTAGTGGTTGGGTTCCCCCTTGTGTGACTAACCATTAAMCACTCCGCCGGGGGAGTCSASCGCAAGGGTGAAAC
TCAAARGAATTGAGGGGGGCCCRCACAAGCGGKGGAGCATGGGGTTTAATTCRAARCAACSCRAAAAACCTTACCA
AATCTTGACATCCTTTGACCACTCTAAAKATAGATTTTTCCCCTTGGGGAGAMAAAATGAGGGGGGGSGKGGTGTT
CTCCYCCTCTCGTGGCGAGATATGGGGTGATACCCCCCCCCAACCGCCCCCCTTTTTATTTTTTTTCCCCWTTTTT
TTGGGGGCTCTCTTGGGTGAGCGGGGGAAAAAAMRGAGAAGGGGGGGGAGAASAMCAAAAAATCTTGCCCCTTATA
ATATGGGGGCCACACMTGTTATAARGGGGAAAAAAAAGGGGGGCGAYACCCCGGGGGGTCCCCCACACCCCAAAAT
TTTTTTCTTTTTTGATATTGTTCTCCGCCTCTYCAGCAYGARGATCTGGTATCTATTATTCTCGAAAAYCRCGTGG
KGRGAKAAMWTTYCCSGKKTTKTGAMACCCCSCSYCTCACMAGAGAGWKTGTSWMACCCCAMMSSGGGAGWAYCYY
TTTTRGMGMCCCSCCAAGGKGGGSRAAWAWTGTGGKGRTGTCTACAASGKCAYCAACAAAAAA

>_isol_47
TGTGGTTGTTCCCTTGCAGGACGTMTTCCTGAGCTAAMGCGTTAGTCSACCGCCTGGGAGACAGTGCAAGACAAAA
ATTAAATGAATTTGAGGGGGGCCGACAAACGGKGGAASATGTGGTTTAATTTCATGCMACGCGAAAAAACTTTACT
ACTCTTGGAMTCCCCAKTAATTACTGTARATGYTTTGKTTCCCCTTCGAACTCTGAGTGAGTGCTGCATGGCTGTC
GTCRKCTCGTGTTGTGAAATGATGTTGTAAKTCCCGCRCRAGCGCAACCCTTATCCTTTGTTGCCGCCGGTCCGGC
CGGGAACTTCAGGATGACTGAGTGATAAACTGGAGGAAGGTGGGGATGACATCWAGTCATCATGGCCCTTATAAGA
CCTGSTACACACGTGCTACAMTGGGRTATACAAARASAAKTGACCTCTCGAGAGARGGAGSACCTCCTAWARWGTG
GCTCACWCCGGATTGTGKTCTGCGMCTCTCCTCCATGTGAWCSGAATCTCTAGWAATCKCASATCTCAATGTGMCG
GTGAATATACTCCCCGGCCTTGTACACACCGCCCGCCACACCATGSGAGTGKKTTGCACAASAARWAGGTAGCTTA
ACCTTCTTTGGGGCGCTTACCMCTTTGTGATTCATGACTGGTGGTGAAGACGTARCAASRGKAWAC

>_isol_49

GTAAKGTTRTGTCCTTTRAGAATGGCYTCCSGAACTAACSCSTTAAMTCCACCSCCYGGGGAGTACCGCCSCCARG
GTAAAACTCAAATGAATTGRCGGGGGCCCSCMCMARCSGTGGARCMTGTGGTTTAATTTCATGCCACSCSAAAAAC
CTTACCTGGTCTTGACMTCCACARAAMTTTYCAAAAATGGRTTGGTGCCCTCMGGAACTGTTAAACARGTGCTGCM
TGGCTGTCCTCCMCTCSTGTTGTKAAATGGTTGGTTTAMTCCCSCMACCARCSCCACCCTTATTCTTTGTTKCCMG
CSGTTCCGCCSGGAACYCMAARGAAACCGCCMRTGAAAAACTGGAAGAARGGGGGGAAGACCTCMARTCMTCMWGG
CCCTTACCACCSGGGCCACCCMCSTGCTAAAATGGCSCAAAAAAAAAAAAACCAACTCCCSARAACCARCCGACCT
CAWAAAGTGCCTCCTAATCCSGAATGGAATCCGCCACYCCACTCCMWGAARTCCGAATCCCTAATAATCCTAAAAC
AAAATGCCACCGTTGAWTWCTTCCCCGGCCTTGTACCCACCCCCCCTCCCACCMTGGGAGTGKGTTKCAAAARAAA
TAWGTARCTTWAYCYTYCGGAGGGCSCYTACCMCTTTGTKATTYATKRCTGGGGTGRASTCCYAACSARGTWAAAC
C

>_isol_50
AGTGGCCTTGAGGTGGCTTCCSGAATACGCSTTAASTCCACCCCCTGGGGAGTACSGSCSCMAGGKTAAAACTCAA
ATGGATTGRSGGGGGCCCGCACAAGSGGGGGAGCATGGGGTTTAATTYSATGCAACSCSAAAAACCTTACCTACTC
TTGACATCCAAAAAACTTTYCARAAATGGATTGGTGCCTTCGGGAACTCTGAAACRGGTGCTGCRTGGGTGTCCTC
MSCTCGTGTTGWGAAATGGTGGGTTAAMTCCCSCMACCARCSCCACCCTTATTCTTTTTGCCMGCKGTTAMGGCG
GGAACTCAAARGARACCGCCMGTGAWAAACTGGAAGAAGGGGGGGAAGACSTCMARTCMTCRTGGCCCTTAASAAT
ARGGCTACCCMCGTGCTACMATGGCSCAWAAAAARAAAAACCACCTCSCGAGAACAAGCSGACCTCAWAAAGTGCS
TCSTAATCCGGATTGGARTCCGCMACTCCACTCCMWGAARTCMGAATCSCTAATAATCSTAAATCAAAATGCTACS
GTKAATACCTTCCCGGGCCTTGTACACMCCSCCCSTCACACCRTGGGAGKTGGTTGCAAAARAARTWAGTWKCTTA
AYCYTYGGGAGGGCKCTTACCWYTTTGTTAWTYAWGASTGGGGTGAASTCMTAACRARGSMAACC

>_isol_26
GATGTGTTAGGGGTTTYCKCCCYYTAKTGCYGCCWCYAAMSCATTAAMCACCCCCCCGGGGGAKTACGGTCCCAAG
AAYGAAACCCAAAGGAATTGGRGGGGGCCCCCCCAARCGGGGGARCMTGGGGTTTAATTYMAAMCAACCCCAAAAA
CCTTACCMGGGCTTGACMTCCYCYGACAACCCWAAAAATARGGCTTTCCCCTCGGGGACAAAATGACAGGTGGTGC
ATGGTTGTCCTCCMCTCCTGTCSTGAAATGTTGGGTTAAMTCCCSCAACCARCSCCACCCCTTWTCTTTWTTTCCA
ACCTTTTTTTGGGCCCTCCAARGGGACCGCCGGTGAAAAACCSGAAGAAGGTGGGGAAGAMSTCMAAACMTCMYGC
CCCTTATKACCTGGGCYACACACSTGCTACAAWGGAAAAAAAAAAARGGGTGCAAMAACSSGAGGTTTAACCCAWCC
CAWAAATCTGTTCYCAATTTSGAACSYAATCCGCMACTCCACTGGGTGAAACTGGAAACCCTAATAAMCCCSGAAC
CACCRGGCCCGGTTAAAATYTTTCCCGGCCTTTTAMACMCCCCCCCTCCCCACASGAGKWTTTCMACACCCCAAA
TCCGTGAAGTAACCTTTAWGGARCCCCCCCCCCAAAGGGGGGMAGATKKATGGGGGGGGAMTCCYAAMSAAGWAAC
AAC

>_isol_45
AAGTGTCATGTGTTRGGGSCCTWCCCTTTTAGTKAAATTCACATTAARCWCTCCCCCGGGGGAGTACGGGCSCAAG
GSTGAAACTCAAAAGAATTGASGGGGGCCCSCACAARCYGTGGAGTATGGGGTTTAATTCAAAACAACSCRAAAAA
CCTTACCAAGTCTTGACATCCCYMAAAAACCCMAAAAAAAAGGCGTCCCCTTCAGGAAAAAAATGACRGGTGGTGG
ATGGTTGTCCTCCSCTCGTGTCGKGAGATGGTGGGTTAACTCCCMCMACCASCRCCCCCCTTTATTTTTTTGCCA
TCATTRTKTTGGGCCCTCTRARGATGCCGGCGGGGAAAAAACCGAAAGAAGGGGGGAAAAACWAAAAATCTTCCCC
CCCTTAAAAACTGGGCCACCCCCSTTCCAAAAAGGAGGGGAAAAARAGGTACAAAACCCCGGGGGGGAAATCATCT
CAAAAAAACCTTCTCTYTTTTTATTGTGGGGGGCCACTCCCCCCCMTGAAAGTGGAAACTTTAKTAATCCCGGGAC
AMCMCGCCGCGGWGAAAATTTTTCCCGGTGTTGTKMACACCCCCCCCTCMCACCACRARAKTTTTTAACMCCCCAAR
TCKGKGGGGKRAMCTTTTTTRGRGSCCSCCCCCCAARGGGGGRSAGAATAATGGGGKGGAGTCMTAASRRRGKMAS
AACA

>_isol_32
KTMTCWTGTGTTTGGGSCCCCTCCCTTTYGTKATYTTCAYWTTAAMCWCTCCCCCGGGGGATCGGKCSCAGASTGA
ATCTCAAAAGAATTGAGGGGGGCCCRCACAARCYGTGGAGTATGGGGTTTAATTCAAAACAACGCRAAAAACCTTA
CCRKGTTTTGATATCCTTTTAAAACCCTAAAAATTGTTTTTTTTCTTCGGGGACAWAAAGAGRGGTGGTGGGTGGT
TGTCCTCMYCTCGTGTCGKGAGATGGTGGGTTTACTCCCRCMAMSAGCACCACCCTTTATTTTTTTGCCCACTTT
TTGGGGGGCCCTTCRAGGGTGCCGGCCGGGAACAAACCGAAAGAGGGGGGGAARAACWAAAAATCTTCCCCCCCTT
AACAACCGGGCCACACCCGTTACAAAAGGGGACAAAAAAAGGGGTTGAAAACCCCGGGTGTTAAACCACTCCAAAA
ATCTGTTCTTTTTTTAGAATTGCATCCGCCACTCCGCCTGMAAAAAGTGGGATCTMTTATTAACCGAYAACCARCC
GGCCCGGATAAATTTTTTCCCGGTTTTTGAACCCCCCCCCCKCCCAACCMRARTATTTTWWACCCCCCRASTTSGG
GGGGKMAMCYTTAARGAMCCCSCCSCMSAARGKGGGRAAKATTAKTGGGGWGAASTYMAAMGGKGKAAAC

>_isol_41

AAGTGTGTTAGGGKTTTCATACCCYTTGTGCCCAAATTTAACAWTTAACCATTCCCCCGGGGAATTACGGCCCCAR
AAYKAAACCTCAAAGGATTTGASGGGGCCCCCACCAACCRTGGAATAAGGTGTTTTAWTTCAAACCACCCCRAAAA
ACCTTACCAGGTCTGGMCTCCCSAWGAMCCCCSMAAAAATKKGGCTTTCCCTTCGGAACMTAAARAACRGGGGTTG
CTGGTTTTCCTCCMCCTCTGGCCKGARAATTTTGGTTTAATTCCCCCACCAACCCCACCCYTTGTCCTTATTTSCM
RCCATTTYKGTGGGCMCYYYWAGGGGAACGGCGGGGAAAAACCGGAAGAAGGGGGGGAAKAASTCCAAACATCMKG
CCCCTTAWGACCGGGGGTACMCCMKTTATAAAATGGCCSGAACAAAGGGCTGTWAAACCCCAAGGGTGAACCAAAC
CCAAAAASCCGGTCCTAATTTRAAAGMAAGGCTACACCCCCCTTCKTGAAWTTAAAAATTCTAACTATCCCGGAAY
ARCSCSCCCCCCGRAAAATCCTTTCCGGGGCTTTTAAACACCCCTCCCTCCCCASMRAAAAKKTTTWAACCCCCAA
ATCCKGGKGGGAAAMYACMRAACCCCACCCCCCAAGGKGGKAMYATRAAKGGGGGKGAATYCMTAWSAAGRAAAAA
CC

>_isol_28
TAAAWGTGTATAGGGGTTTCCKCCCCYTWATKSYKCMRCYAACSCMWTAAACACYCCCCCCGGGGAGTACSGGCCC
MAGAAYGAAACYCAAARGAAWTTGRSGGGGCCCCCACMARCCGGGGAACATGGTGTTTAATTTCAAACCACCCCAA
AAAACTTTACARGGCTTGACMTCCTCTGACMACCCTAAAAATARGGCTTTTCCTTTSGGGACARAATGACMRGGGG
TGGCTGGTTGTCCTCCSCTCCTGTCCKGAAATGGTTGGTTTAATTCCSCMACCAACCCCACCCTTTATCTTAWTTT
CCCACCTTTYRTTTGGCCCTCYAARGGGACCGGCGGTTGAAAAACSGGAGAAGGGTGGGAAKACSTCMAATCCTYC
YGCCCCTTAAKAACYGGGCCACCCMCSTTCTAAAATGGAAAAAAAAAAAGGGCTCCAMACCSCSAGGGTTTACCCA
TCCCAWAAATTTGTTTYKWWTTTSGGACCSAATTCGCCACTCCACTGGGTGAAACTTGAATCCCTAATAATCCCCG
ATCCRCATGCCCCGGTTAAAAACTTCCCSGGCCTTGKAACCCCCCCCCCTCCAACCMAAAARTTTTCCACCACCCA
AATTCGTGAAGWAACCTTTAARGRMCCCSCCCRMAAAGGGGGGGSAAAAAAAATTGGGTTAAATTCWAAAAAAGGM
AACC

>_isol_04
AAGTMTTAGGTTWGGGTTTCCCCCTTGTGCTKAACYTCAWTTAACWCTCCGGCTGGGGAGTCCGGCGARAGAAGAA
CTAAAAGAATTGGGGGGCGCCCRCACAACYGTGGAACATGGGGTTTAATTCAAASCAACSCAAAAACCTTACCRWG
TTTTGATATCCTWTTKAACCCYTAAAAAATRKTTTTCCCCCTTGGGGGAARAAGGAGAGGGGGGGGGKGGTTGTCY
YCYCCTCTTGTGKYGAGATGTGGTGTTAAATCCCCCAAAAAGCGCCCCCCCTCTTTTTTTTTTCCARCATTTTTTT
GGGGCCTCTAAAGGGACCGCGGGTGAAACAAAMGAAGAAGGGGGGGAGAARAMCTAATCTTCYCCCCCCTTATAAG
AGGGGGTACACACSTGCTATAATGGGGASAAAAAAAGGGGRARCAACCCCGCGGGGTYGACCCCACCCCAAAAATC
TTTTCCCTTTTCGAATTGMGGGGCYCCACCGCCGTGAGKAAAAGGAAAATCTAATAATCSCMGAACARCSCGCCGC
GGAGAAAATTCTCTCGGGTGTTGTACACACCCCCCCTCMCACCACRAGAGTTTTTAACCCCCCCMMYCSGKGAGGW
AAWWCTTTAGAGMCCCCCCCCWAARGGGGGRAAKATAATTGTGGGGAKRTCTTMAMARGGKRAACA

>_isol_09
WAAAKGTGTTAGGGGTTTCCKCCCCYTWATKSYKCCACYAACSCMWTTAACACCCCCCCCGGGGAGTACCGGCCCC
ARAAYGAAACTCCAAAGAATTTACSGGGGCCCCCACMARCCGTGGAACATGGTGTTTAATTTCAAACCACCCCAAA
AAACCTTACMRGTCCTGACMTCCTCCGACAACCCCAAAAAAWAGGCTTTCCCCTTCGGGAACAAATTACCRGGGGT
GCCTGGTTGTCCTCCRCTCCTGTCCKGAAAAGGTTGGTTTAATCCCCCCACCAACSCCACCCTTTATCCTTATTTC
CMACCTTTWRTTTGGCCCTCYAARGGTAACGGCSGTGACAAAACSGAAGAARGGGGGGAAKAACTCCAATCCTCCW
GCCCCTTAAKAACYGGGCCACCCMMCTTCCACMATGGAAAAAAAAAARGGGTGCCARACCSCMARGGTTAACCCAT
CCCAMMAATTTTTTCYTYATTTRGAACCSAATCCGCCACTCCRCCGCSTGAAACTTGAATCCCTTATTATCCCCGA
ATCACCTGGCGCCGTTAAAAYCTTTCCSGGKCTTGTACACCCCCSCCCTCCCAMCMMSARAKTTTTCCACCCCCSA
ARTTCGTKAAGTTAYCTTTAARGMACCCRCCCCCCAAAGTGGGGGSYAATTAATGGGGTGGAATTCTAARMAAGGA
AAACC

>_isol_07
TACATGTTAGGAGTTKTTCCMCYTTAWKSCTCCCYAAAMGTTTAACACYYCCSCYGGGGAAAAAAGACGCAAAGGT
TAAAACYCAAAGGATTTGASGGGGCCCCCCTCAGCCGGTGAACCWGGTGTTTAAWTTCAAACCACCSCAAAAAACC
TTACCAGWCCTGGACACCCCCGAACCCCCCAAAAAAAAGGGTTTCCCCTTCGGGGGAACATAAWAAGGGGGGGGGG
GTGTTTCTCCCCCCCTCTGGTCGGAAATTGTGGGTTAAMCCCCCACAACCACCCCCCCCTTTWATTTTTTTCCCCA
TTTATATTTGGGCACCAAAAKTTAAACGGGGGGAAAAAACCAAAGAGGGGGGGGAAAAAACKWAAAMTCAYCSCCC
CTTTTATRTTGGGGGCTAACCTGCTACAAGGGGAAAAAAAAAAGGGGGTCCCACACCGGAGGGTTCACCAAATCCA
AAKATTTTTTTCAATTATACAATTTATTTCCCCCCCCCCCAGCC... (a partir 520 nπo leu
mais...)

>_isol_16

AGAATCTTTTGTGGTTTGGCCTCCCCCTTTTGCTGAKCTAACAWTTTAATACTCCGCCTGGGGAGTCCGSCGAMAG
AAAAAAACTCAAAAGAATTGGGGGGCGCCCCCCCAACGGGGGGAGCATGGTTTTTATTTTRTACCAACRCAAAAAA
CCTTACCACGGCSTGACATCCTAAAACTTTCCAAAAAATAGGTGTTTCCCCTYGGGGATCYKAAAGAGTGGTGGGT
TGTTGTCCTCWKCTKTGTTGAAAAGATGTTGGGATTAMTCCCCCAACSAGCRCCACCCTTTTTTTTTTTTTGCCCM
TTTTTTTTTGGGGKACCTAAAAGAAACGGCGGTGAAAAAARWGGAAAAAGGGGGGGAAAAAATWAAATTATCMTGC
CCCTTTTGAAGGGGGGCCACACACGTGCTAYAGTGGAAAAAAAAAAAAGGGGTGCCACCTCCCMGGGTTTAMCCCCC
CCCAAAAATTTTTTTTCTCTTKAGAGATGGATTTCCCACCCCCCCGTCAAGAARGTGGAATCSCTAATAATCGAAS
AACAWAATGCCGCGGTGAATATYTCCCCGGGSCTTGTACACCCCCCCCGTCACACCAMGAGAGTTTGTTRCAMAAR
AARTTSGTGRMRTAACCTTTTRRGGSGGCCCCCCMCCMMATGGGGSRTYAATRATTGGGGGGAATTCWAAMRRGGY
AAAACC

>_isol_35
TTACCGTGTTAGGGGTTTCCCCCCCTTTATGCTTCCCTAAACGTTTAACACTTCCSCCTGGTATAAAGGGCCSARA
AYTAAAACCCAAGGAWTTGMSGGGGCCCCCCAAAACCGTGGAACCTGGTGTTTTATTTCAAACAACCCCAAAAACC
TTTCCCRGGCCTGGATCCCCTTTAACCCTGGAAAAATATTKTTTCCCCCTTCGGGGCCACCGTTAACRGGGGTTGA
TTGTTTTCCTCCSCTCCTGTCCTGGRATGGTTGGTTTAMTCCCCCCACCSSCCCCACCCCTTRTTTTTATTTCCCT
TCTTTTATTTGGCCCCCCRARGGTACCGGCGGTGAACAAACCGAAGGAAGGGGGGATTAACWCCAATTAWCCAGCC
CCTTWTGAACCGGGGCTAACMAGTTCTKRATGGACYAAACCAATTGGTTGCCAACCGCAAAAAGGAACCTATACCA
AT... (a partir 478 nπo leu mais...)

>_isol_56
TAWGAWGTGTTAGGGGKTTCCCCCCCYTWATKSYKSCACYAAASCMWTTAACACTCCCCCCGGGGAATACCGGCCC
CARAAYGAAACYCCAAAGAAATTGMSGGGGCCCCCCCMARCCGTGGAACCTGGTGTTTAAWTTCAAACCACCCSAA
AAAACCTTACMRGGTCTTACMTCCCSTTTAACMMTGGAAAAATAATWTTTCCCCCTTCGGGGCCAASGTTAACRGG
GGTTGCTGGTTTTCCTCCMCCCCTGGCCKGAAATGGTTGGTTTAATTCCSCCACCARCCCCACCCYTTGTTCTTAT
TTCCCTTCTTTTATTTGGCCCTCCAARGGTGCCGGCSGTTAACAAACSGAAGAAAGGGGGGAAGAACTCCAATCCW
CCMGCCCCTTAAKAACYGGGCCAACCCMCTTCTAAAAAGGAASAAACCAAMSGTTTCCMACTCSSSAAAAGGAACC
AAWCCCATWAAATTCTTTTCMATTTCGAATTGAAGCCKCCACTCCCCCACMAKAAACCSGAAACCCTAATAATCCS
SGAACCACCAGCCSCSGWTAAATACTTTCCSGGSCCTTKACCCCCCCCCCCTCCCACCMASARAATTTKTTACACC
CCSRARTCCGTKAAGTWACCCTTTTKGAACCCACCCCCCAAAGGKGGAAAAAATTAWTGGGGKGRAA-
TCCTAAMMAAGTAAACC

- - - - - - - - - - - - - - -
